# Neurocognitive mechanisms of source credibility: how work experience and patient ratings modulate the processing of medical veracity cues

**DOI:** 10.3389/fnhum.2026.1867901

**Published:** 2026-09-18

**Authors:** Eliana Monahhova, Alexandra Morozova, Julia Gorodnicheva, Semyon Mening, Oksana Zinchenko, Vasily Klucharev

**Affiliations:** 1 Centre for Cognition and Decision Making, Institute for Cognitive Neuroscience, HSE University, Moscow, Russia; 2 International Laboratory of Social Neurobiology, Institute for Cognitive Neuroscience, HSE University, Moscow, Russia; 3 Laboratory for Cognitive Research, Faculty of Social Sciences, HSE University, Moscow, Russia

**Keywords:** source credibility, expertise, trustworthiness, fakes, opinion change, persuasiveness, medicine, EEG

## Abstract

**Inroduction:**

Source credibility is fundamental to how medical information is processed,yet the underlying neurocognitive mechanisms remain poorly understood. We investigated how two credibility cues — a doctor’s expertise (years of work experience) and aggregate patient ratings (star ratings) — modulate brain responses to veracity cues (‘True’ vs. ‘False’) presented after health- related headlines.

**Methods:**

We recorded EEG from 36 participants while they evaluated true and false headlines across various medical topics (e.g., vitamins, GMOs, vaccination). Each headline was paired with a fictional doctor’s profile that systematically varied in expertise (work experience) and patient ratings (star ratings).

**Results:**

Behaviorally, while both doctors’ characteristics predicted opinion change and persuasiveness of veracity cues, patient ratings exerted a significantly stronger effect than work experience only on the persuasiveness of veracity cues. Critically, ‘False’ cues elicited a significantly stronger FRN(RewP)-like response when delivered by highly rated doctors compared to less rated ones; no such modulation was observed for ‘True’ cues. Source localization using standardized low-resolution electromagnetic tomography (sLORETA) identified frontal and posterior medial cortices as the likely neural generators of this effect.

**Discussion:**

Overall, our findings suggest that source credibility cues modulate the effectiveness of medical veracity feedback, revealing distinct neurocognitive mechanisms that underpin how credibility differentially shapes the processing confirmatory and disconfirmatory cues.

## Highlights

- Both years of work experience and patient ratings significantly predicted the behavioral impact of doctors’ veracity cues.
- Patient ratings shaped perceived persuasiveness more strongly than doctors’ work experience.
- Veracity cues ‘False’ elicited a stronger FRN(RewP)-like response when delivered by doctors with higher patient ratings.

## Introduction

In an era characterized by unprecedented access to information, individuals face the challenging task of distinguishing credible from non-credible sources, particularly in domains with significant personal impact such as healthcare. Source credibility has long been recognized as a fundamental concept in communication research that plays a critical role in determining how persuasive messages are perceived and internalized by audiences (Hovland and Weiss, 1951; Whitehead, 1968; Pornpitakpan, 2004). In medical contexts, false information from seemingly credible sources can lead to harmful health decisions and outcomes (Wang et al., 2019; Kington et al., 2021). Consequently, doctors play a pivotal role in combating medical misinformation. This challenge is particularly pronounced in online health environments, where users routinely rely on heuristic credibility cues — such as author profiles, badges, and aggregated ratings — to evaluate medical sources at scale (Metzger, 2007; Metzger and Flanagin, 2011). The current study examines the impact of veracity cues provided by doctors on the persuasiveness of medical misinformation, specifically how this persuasiveness varies depending on the source credibility cues (work experience and aggregate patient star ratings). Additionally, we investigate the neurophysiological correlates underlying the processing of these cues across varying levels of credibility.

Source credibility is often viewed as a dual-dimensional construct consisting of expertise and trustworthiness (McGinnies and Ward, 1980; Wiener and Mowen, 1986; Pornpitakpan, 2004). Expertise refers to the perceived capability of a communicator to make accurate assertions, while trustworthiness pertains to the audience’s perception that the communicator believes in the validity of their own statements (Hovland and Weiss, 1951). Many studies have proposed additional dimensions of source credibility, such as dynamism (Berlo et al., 1969), authoritativeness, character (McCroskey, 1966) and objectivity (Whitehead, 1968). However, the precise factorial dimensions of source credibility remain a subject of ongoing debate (Pornpitakpan, 2004; McCroskey and Teven, 1999; Hanimann et al., 2023). Consequently, we examined the electrophysiological signatures of source credibility during the processing of veracity cues provided by doctors for medical misinformation, focusing specifically on both perceived expertise and rating-based credibility.

The ability of source credibility to boost persuasion is a robust finding, documented across decades of behavioral research. Highly credible sources are typically more persuasive than those with low credibility (e.g., Hovland and Weiss, 1951; Horai et al., 1974; Heesacker et al., 1983; Pornpitakpan, 2004). The persuasive effects of source credibility have been extensively examined within theoretical frameworks such as the Elaboration Likelihood Model (Cacioppo et al., 1986) and the Heuristic-Systematic Model (Chaiken, 1980). These models posit that source credibility serves as a peripheral cue or heuristic when cognitive elaboration is low, or as a factor that enhances message scrutiny when elaboration is high. A meta-analysis by Kumkale et al. (2010) has further confirmed that persuasive effects of source credibility are robust across diverse contexts, though their magnitude varies based on message relevance and recipient involvement.

Yet, scholarly debate persists regarding which dimension of credibility exerts a dominant influence on persuasive outcomes. For example, results of Hovland and Mandell (1952) suggested that a perceived low level of trustworthiness does not necessarily reduce persuasiveness. However, McGinnies and Ward (1980) revealed that the trustworthy communicator was more influential than the untrustworthy one, whether or not he was an expert, particularly in the context of political persuasion. In clinical and public health contexts, a more granular understanding of the behavioral effects of source credibility is particularly paramount. The meta-analysis of 20 studies by Yang and Beatty (2016) indicated that expert sources have a stronger influence on perceived credibility in online health contexts compared to offline environments. The findings suggest that consumers weigh expertise more heavily when evaluating source credibility in increasingly dominant digital environments. Furthermore, Liu et al. (2022) demonstrated that in the context of health chatbots, source expertise acts as a critical heuristic cue that effectively increases usage intentions. In contrast, Lederman et al. (2014) found that when medical information is anonymous, users primarily judge credibility through social cue signaling ‘crowd consensus’.

The current study investigates the persuasive effects of medical doctors’ source credibility. Specifically, we systematically manipulated expertise — operationalized as years of work experience, a core marker of professional authority (Ohanian, 1990) — and the aggregate patient star rating, which serves as the multi-dimensional peripheral trust cue most commonly encountered on online medical platforms. Importantly, within the broader literature on digital health communication, ratings are increasingly recognized as compound signals that reflect dimensions adjacent to trustworthiness, including experiential credibility, perceived reputation, and social endorsement (Claggett et al., 2024; Hocevar et al., 2017; Metzger, 2007; Metzger and Flanagin, 2011). For instance, Han et al. (2021) showed that while consumer trust fully mediated the effect of review volume on doctor selection, it did not mediate the effect of the numerical rating. Rather, the rating’s impact was conditional upon the reader’s prior trust and regulatory focus. Acknowledging this complexity, we operationalized patient ratings as a multi-dimensional, ecologically valid credibility cue. Overall, this study design allowed us to investigate the electrophysiological signatures of veracity cues provided by doctors with varying levels of credibility.

Neuroimaging studies have uncovered some neural signatures of cognitive processing of source credibility. For example, a seminal study demonstrated that in the context of social exchange, the caudate nucleus encodes trust-building signals during iterative economic interactions (King-Casas et al., 2005). A coordinate-based meta-analysis of 30 fMRI studies demonstrated consistent activation of the anterior insula during trust decisions in reciprocity paradigms (Bellucci et al., 2017). Although a substantial body of fMRI work has also linked the amygdala to automatic trust evaluation, specifically from faces (Winston et al., 2002; Adolphs et al., 1998), source credibility based on expertise and reputation appears to recruit a partially distinct network centered on the caudate nucleus, hippocampus, and prefrontal cortex (e.g., Klucharev et al., 2008; King-Casas et al., 2005). Despite this growing body of neuroimaging evidence, EEG studies of source credibility remain comparatively scarce. Kawiak et al. (2020a), Kawiak et al. (2020b) conducted pioneering electrophysiological research in this domain, revealing significant differences in event-related potentials (ERPs) between trusting and distrusting decisions in two relatively late time windows (268–300 ms and 372–796 ms). Source localization analyses identified the anterior cingulate cortex, insular cortex, and subparietal sulcus as generators of these credibility-sensitive responses. Interestingly, machine learning classifiers trained on EEG data achieved over 70% accuracy in distinguishing ‘reputed’ from ‘earned’ source credibility (Kawiak et al., 2020b).

Converging evidence from related fields further supports the view that the brain processes information differentially depending on source credibility characteristics. Notably, Edelson et al. (2011, 2014) demonstrated that social influence distorts memory traces via enhanced amygdala-hippocampal connectivity, with subsequent prefrontal correction mechanisms engaged during recovery from misleading influence. Complementing this, Kwasniewicz et al. (2021) showed that evaluating message credibility without prior knowledge of its source activates neural regions associated with uncertainty. Additionally, Schneider et al. (2022) demonstrated that earned source credibility evaluation recruits distinct brain networks compared to message credibility processing, confirming that source-level and message-level evaluations involve separable neural mechanisms. To our knowledge, it remains unexplored how expertise-driven versus aggregated patient rating-driven credibility dimensions modulate the neural processing of veracity cues (e.g., fact-checking labels), particularly within the context of medical communication. To address this, we utilize ERP methodology to capture the fine-grained temporal progression of how work experience and patient ratings influence the neural evaluation of information veracity.

The current study utilizes EEG to examine the feedback-related negativity (FRN)—a frontocentral negative deflection occurring approximately 200–300 ms post-feedback. The FRN, often discussed in the context of the reward positivity (RewP), is sensitive to the valence and expectancy of feedback. The FRN is enhanced when feedback contradicts expectations or prior beliefs (Hajcak et al., 2007) and has been linked to salience prediction errors, where it responds to unexpected events regardless of valence (Talmi et al., 2013; Hauser et al., 2014). The same deflection is often described as the RewP when framed around favorable, rewarding outcomes (Foti et al., 2011; Holroyd et al., 2011; Proudfit, 2015). Specifically, we hypothesized that veracity cues from credible versus non-credible doctors would modulate the FRN(RewP) component, indicating that source credibility serves as a prior that guides the neural evaluation of truth-related feedback. Previous studies suggest that the FRN(RewP) signals a need for behavioral adaptation and helps individuals update their internal models to improve future decision-making (Holroyd and Coles, 2002; Hajcak et al., 2007; San Martín, 2012). While the FRN(RewP) has an established role in reward processing (e.g., Glazer et al., 2018), we argue that it also functions as a sensitive neural marker for evaluating the reliability of corrective information provided by different sources.

In the current study, participants were presented with health-related headlines followed by veracity cues (‘True’ or ‘False’) attributed to medical doctors with varying levels of work experience and patients ratings. These cues indicated whether the doctor categorized the headline as accurate or false. We hypothesized that cues from more credible doctors would trigger a stronger revaluation of the headlines compared to those from less credible sources. Accordingly, we expected highly credible sources to evoke a more robust FRN(RewP)-like responses to veracity cues. Overall, we hypothesized that veracity cues from highly credible doctors would lead to greater opinion change regarding medical headlines, higher perceived persuasiveness and increased FRN(RewP) amplitudes compared to veracity cues from doctors with lower credibility. Furthermore, we investigated whether individual differences—specifically higher levels of analytical thinking, need for cognition, and conformity—were associated with a greater reliance on veracity cues provided by high-credibility doctors.

## Materials and methods

### Methodology

The experiment consisted of two EEG sessions (Experimental Session 1 and Control Session 2), followed by a post-experimental behavioral session in which participants completed several questionnaires. During EEG Session 1, each trial required participants to evaluate a medical headline before being exposed to a veracity cue (‘True’ or ‘False’). At the end of each trial, participants were given the opportunity to reevaluate the medical headlines. During this session, we recorded and analyzed ERPs time-locked to the veracity cues provided by credible and non-credible sources. In the control EEG session (Session 2), participants performed a simple behavioral task in order to record standard feedback-related ERPs. Upon completion of the study, participants filled three questionnaires: the Cognitive Reflection Test (CRT), the Need for Cognition Scale (NFCS), and the Conformity Scale (CS). All stages of the study are described in detail in the *Stimuli* and *Procedure* subsections. The study was approved by the ethics committee of HSE university and conducted in accordance with the Declaration of Helsinki. All participants provided written informed consent prior to their participation.

### Participants

We recruited 43 right-handed adult participants through open invitations in local communities and social media platforms. We excluded 7 participants from further data analysis due to extensively noisy EEG recordings, based on the standard artifact rejection criteria. The final dataset consisted of 36 participants (28 females, aged 18–35, mean [*M*] age = 20.4, standard deviation [*SD*] = 1.78). All participants had normal or corrected-to-normal vision, either studied at a university or had completed higher education and reported no history of neurological or psychiatric disorders. Importantly, participants confirmed that they had no educational background connected with medicine and were not employed in a medical-related field. After the participants completed the experiment, they received 750 monetary units, equivalent to approximately 26 USD, when calculated based on purchasing power parity (Organisation for Economic Co-operation and Development [OECD], 2023).

### Stimuli

The stimulus set included 120 health-related headlines (statements) reflecting different topics regarding the medical field, including vaccines, vitamins, genetically modified organisms (GMOs), lifestyle and alternative medicine. The headlines have been previously validated in two dimensions: veracity (true vs. false) and valence (positive vs. negative; Artemenko et al., 2023). For example, within the ‘vaccines’ category, participants were presented with headlines such as (translated from original language): ‘All vaccines are hypoallergenic’ [false, positive valence], ‘Many infections can spread regardless of how neat we are’ [true, negative valence]. A complete list of headlines is provided in Supplementary Table S1.

We created profile cards for 19 fictional doctors that displayed their initials, work experience (represented in years), and patient trust ratings (represented by star ratings). Participants were explicitly instructed that star ratings represented aggregate patients’ assessments of each doctor’s trustworthiness, as commonly displayed on online medical review platforms. Sixteen of these doctors varied systematically across credibility dimensions, while three additional doctors served as fillers to mask the experimental design. To avoid introducing gender as a confounding variable, all doctor profiles were presented as male with male initials. To create four experimental conditions, the two dimensions were operationalized at two levels: work experience (high: 18–22 years vs. low: 1–5 years) and patient ratings (high: 4–5 stars vs. low: 1–2 stars). Intermediate levels of work experience (8–12 years) and patient ratings (3 stars) were used to create filler trials, which were subsequently excluded from the statistical analysis. The veracity cues were presented as binary evaluative labels (‘True’ or ‘False’), indicating the fictional doctor’s assessment of the medical headline’s accuracy. Critically, to ensure the credibility manipulation was not confounded with headline’s veracity, the doctors’ cues always accurately reflected the pre-defined truthfulness of the headlines across all trials.

## Procedure

### Experimental EEG session 1

First, participants were exposed to medical headlines (see Supplementary Table S1) and indicated their level of agreement with each headline on a 7-point Likert scale (1 = ‘absolutely disagree’ to 7 = ‘absolutely agree’) presented on the screen. After the evaluation of a headline, a fixation cross appeared on the screen for 1,000 ms, followed by a doctor’s profile card, which was presented for 5,000 ms. The profile card displayed the fictional doctor’s initials (ex., Trifonov M. P.), work experience (represented in years) and patient rating (represented in stars). Next, after a fixation cross with a variable duration from 250 to 500 ms, the doctor’s cue, either ‘True’ or ‘False’, was displayed for 1,500 ms.

After observing the cue, the fixation cross appeared again with a variable duration from 250 to 500 ms and participants could re-evaluate the headlines using the same 7-point Likert scale (i.e., *opinion change ratings*). Finally, participants rated how persuasive (i.e., *persuasiveness ratings*) the doctor’s cue was on a separate 7-point scale (1 = ‘absolutely non-persuasive’ to 7 = ‘absolutely persuasive’). Hereafter, *opinion change* refers to (1) an absolute difference between a participant’s initial and post-cue evaluations and (2) a signed, direction-aware measure, calculated as the post-cue reevaluated opinion minus the initial opinion (see details in *Statistical analyses of behavioral data* subsection), and *persuasiveness* denotes the participant’s evaluation of how persuasive the doctor’s veracity cue was. The rating scales were self-paced, with no time constraints imposed on participant responses. Health-related headlines, doctoral profiles, and veracity cues (‘True’/'False’) were counterbalanced across participants to control for order effects (see Figure 1 for schematic representation of the trial structure).

**Figure 1:**
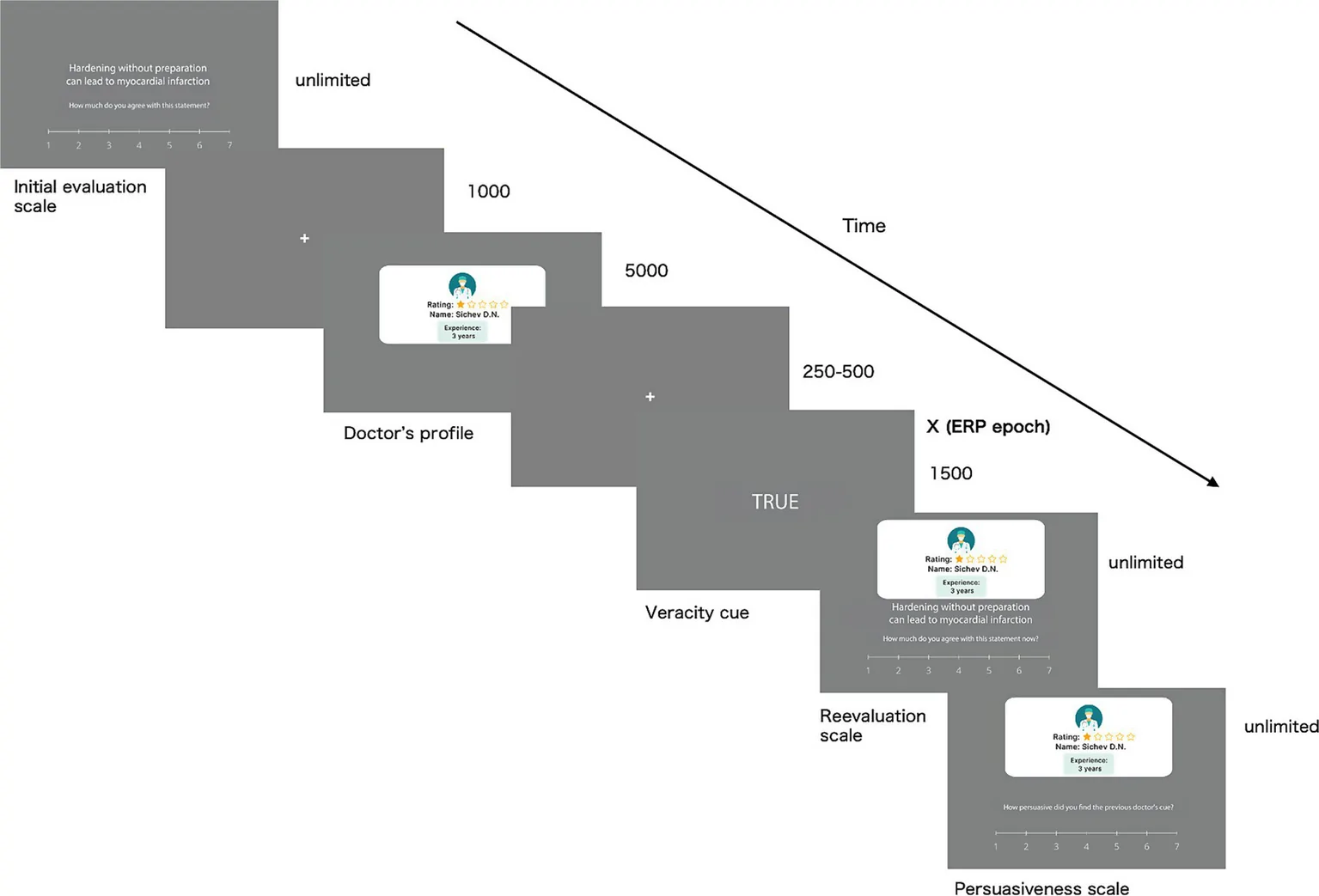
Persuasion task. Schematic representation of the trial structure in EEG session 1. Participants first evaluated health-related headlines on a 7-point Likert scale, followed by a doctor’s profile card displaying initials, work experience, and aggregate patient rating. Next, a veracity cue (‘True’ or ‘False’) appeared on the screen. At the end of the trial, participants were able to re-evaluate the headline and rated persuasiveness of the veracity cue.

### Control EEG session 2

During a control EEG session 2 the same subjects participated in a simplified time estimation task (adapted from Kirsch et al., 2022). In each trial, participants estimated the duration of a one-second (1,000 ms) interval by clicking the mouse button when they felt one second had elapsed. The time window for correct responses was adapted depending on the success of the experiment. The computer algorithm increased the response time-window by 20 ms after error trials and decreased by 20 ms after correct trials symmetrically. Positive feedback for a correct response was given in the form of a green smiley, negative feedback for an incorrect response was visualized with a red frowny, presented on the screen for 350 ms. The task consisted of 350 trials in total.

### EEG recording and processing

EEG data were recorded from 64 active electrodes (actiCap, Brain Products, Germany) positioned according to the standard 10–20 system. Two additional electrodes placed at the mastoids (TP9 and TP10) served as the online reference. Eye movements were detected via HEOG and VEOG electrodes located on the lateral corner of the left eye and under the right eye. The data were digitized at the sampling rate of 500 Hz, low pass filter of 250 Hz. Electrode impedances were kept below 10 kΩ throughout the recording.

For both EEG sessions data preprocessing and subsequent analyses were performed using MNE Python (v.1.8.0) in the JupyterLab environment (v3.6.2). First, the data were band-pass filtered (1–45 Hz) and notch-filtered at 50 Hz. Next, we visually inspected the data for bad channels and noisy segments. Bad channels were interpolated based on the signals of the good sensors around them. To attenuate artifacts caused by eye movements and blinks, we performed Independent Component Analysis (ICA) using the extended infomax algorithm (Makeig et al., 1995).

In both EEG sessions, ERPs were calculated by averaging the EEG epochs time-locked to the cue (Study 1) or feedback (Study 2) onset. The continuous EEG data were segmented into epochs spanning from −400 ms to 1,000 ms relative to the cue (Study 1) or feedback (Study 2) onset. Each epoch included a − 400–0 ms pre-stimulus baseline period. The data were baseline-corrected and grand-averaged across all participants.

### Questionnaires

To assess individual differences in personality traits, participants completed three standardized questionnaires after the EEG sessions: the Cognitive Reflection Test (CRT; Frederick, 2005) to measure participants’ level of analytical thinking; the Need for Cognition Scale (NFCS; Petty and Cacioppo, 1984) assessing the level of engagement in effortful cognitive activity; and the Conformity Scale (CS; Mehrabian and Stefl, 1995) measuring individual tendencies toward conforming to group opinions.

### Statistical analyses

#### Statistical analyses of behavioral data

Statistical analysis was performed using R software (R Core Team, 2024), with significance set at *p* < 0.05 for all tests. As our primary analysis, we conducted multiple linear mixed-effects models to investigate the relationship between *opinion change* (or *persuasiveness ratings*) and the variables characterizing both the stimuli and participant personality traits—specifically, doctors’ work experience and patient ratings, veracity cue type, and CRT, NFCS, and CS scores. Opinion change was measured using two distinct metrics: (1) an absolute measure, calculated as the absolute difference between participants’ initial and post-cue reevaluated opinions (|Opinion _post_ - Opinion _initial_|), and (2) a signed, direction-aware measure, calculated as the post-cue reevaluated opinion minus the initial opinion (Opinion _post_ - Opinion _initial_). The latter was utilized specifically to test whether belief updating was cue-consistent (i.e., shifted toward the implication of the veracity cue) rather than reflecting undirected response variability.

Models were fit to trial-level responses using a fully crossed random-effects structure. This included random intercepts for participants, statements (headlines), and doctor profiles to account for non-independence across grouping factors. Following the maximal-structure recommendation (Barr et al., 2013) while retaining a structure supported by the data (Matuschek et al., 2017), we also specified random slopes for experimental factors manipulated within each grouping unit. Specifically, we included by-participant random slopes for cue type, patient ratings, work experience, and their interactions, alongside a by-statement random slope for cue type (as each statement appeared under both ‘True’ and ‘False’ conditions). Doctor profiles, which exhibited a small number of levels and near-zero intercept variance, were modeled with a random intercept only. The random-intercepts and random-slopes structures were systematically compared using AIC, BIC, and likelihood-ratio tests. Finally, model assumptions—including linearity, independence, homoscedasticity, and normality of residuals—were verified using diagnostic plots, and multicollinearity was assessed via variance inflation factors (VIF). To place the two credibility predictors on a comparable scale, each outcome was standardized (z-scored) prior to model fitting, so that the reported coefficients are expressed in standard-deviation units; this linear rescaling leaves the model structure and all significance tests unchanged. The difference in magnitude between the standardized effects of patient rating and work experience was then tested formally with a likelihood-ratio χ^2^ contrast (df = 1).

To complement these trial-level models with a distribution-free, condition-level check, we computed non-parametric analyses. We tested the normality of the data using the Shapiro–Wilk test, then implemented Friedman’s test and conducted pairwise comparisons using paired-samples *t*-tests (for normally distributed data) and the Wilcoxon signed-rank test (for data that was not normally distributed) to examine differences in opinion change and evaluation of persuasiveness between four experimental conditions: high/low work experience × high/low patient ratings. *p*-values from multiple pairwise comparisons were adjusted using the Bonferroni correction. As a distribution-free robustness check complementing the mixed-effects models, these condition-level non-parametric analyses are reported as descriptive, convergent evidence in Supplementary Tables S2, S3.

#### Statistical analyses of ERPs

We tested the hypothesis that veracity cues from highly credible doctors would elicit stronger FRN-like responses than those from less credible sources. First, we performed a cluster-based permutation F-test across all channels to examine the main effects and interaction of the veracity cue type (‘True’ or ‘False’) and doctor credibility (high/low work experience × high/low patient ratings). The test was based on pairwise comparisons between trial types, with a permutation approach (1,024 permutations) used to correct for multiple comparisons. Significant clusters were identified using a cluster-forming threshold of *p* < 0.05 and a corrected cluster-level significance threshold of *p* < 0.05.

We also fitted regression models that included the elicited amplitude as a predictor of opinion change and persuasiveness; these models were fit to participant-by-condition means (averaged within each credibility condition separately for each cue type). For supplementary sLORETA source localization, we calculated the current density distribution using grand-averaged difference maps (high experience/high rating minus low experience/low rating) separately for ‘False’ and ‘True’ veracity cues. Source localization was performed using the sLORETA software and the MNI152 template to estimate the neural generators of the identified effects.

Finally, as a confirmatory analysis, we conducted a three-way repeated-measures ANOVA to test whether ERP amplitudes in the traditional FRN window defined *a priori* from literature (200–300 ms; e.g., Zhang et al., 2012; Zhang et al., 2017; Mei et al., 2018) were significantly affected by cue type (true vs. false), work experience (high vs. low), and patient rating (high vs. low). We specifically examined the main effects and all possible interactions (cue type × work experience, cue type × patient rating, work experience × patient ratings, and cue type × work experience × patient ratings).

## Results

### Behavioral results: persuasion task (EEG session 1)

#### Descriptive statistics

Table 1 and Figure 2 illustrate that participants demonstrated the largest opinion change (defined as the change in agreement with headlines) when exposed to veracity cues from doctors with the highest work experience and patient ratings (M = 0.78, SD = 0.45), and the least change when cues came from doctors with the lowest work experience and patient ratings (M = 0.38, SD = 0.35). Similarly, veracity cues from doctors with the high work experience and patient ratings were evaluated as the most persuasive (M = 5.61, SD = 0.94), while veracity cues from doctors with the low work experience and patient ratings were rated the least persuasive (M = 3.75, SD = 1.21). Overall, participants demonstrated moderate scores for CRT (M = 3.69, SD = 2.18), NFCS (M = 64.44, SD = 9.17), and CS (M = −7.72, SD = 12.83). The corresponding condition-level non-parametric analyses (Friedman tests and Bonferroni-corrected pairwise comparisons) are provided in Supplementary Tables S2, S3.

**Table 1:** Descriptive statistics for opinion change, perceived persuasiveness, and personality traits.

Variable	Mean	Median	SD	Max	Min
Opinion change					
High work experience/ High patient rating	0.78	0.76	0.45	1.67	0.00
High work experience/Low patient rating	0.45	0.30	0.41	2.00	−0.10
Low work experience/High patient rating	0.61	0.54	0.37	1.38	−0.03
Low work experience/ Low patient rating	0.38	0.28	0.35	1.40	−0.24
Persuasiveness					
High work experience/ High patient rating	5.61	5.77	0.94	7.00	1.48
High work experience/Low patient rating	4.19	4.23	1.10	6.43	1.67
Low work experience/High patient rating	4.96	5.28	1.11	6.48	1.60
Low work experience/ Low patient rating	3.75	4.01	1.21	6.32	1.55
CRT	3.69	4	2.18	7	0
NFCS	64.44	63.5	9.17	85	37
CS	−7.72	−7	12.83	22	−29

**Figure 2:**
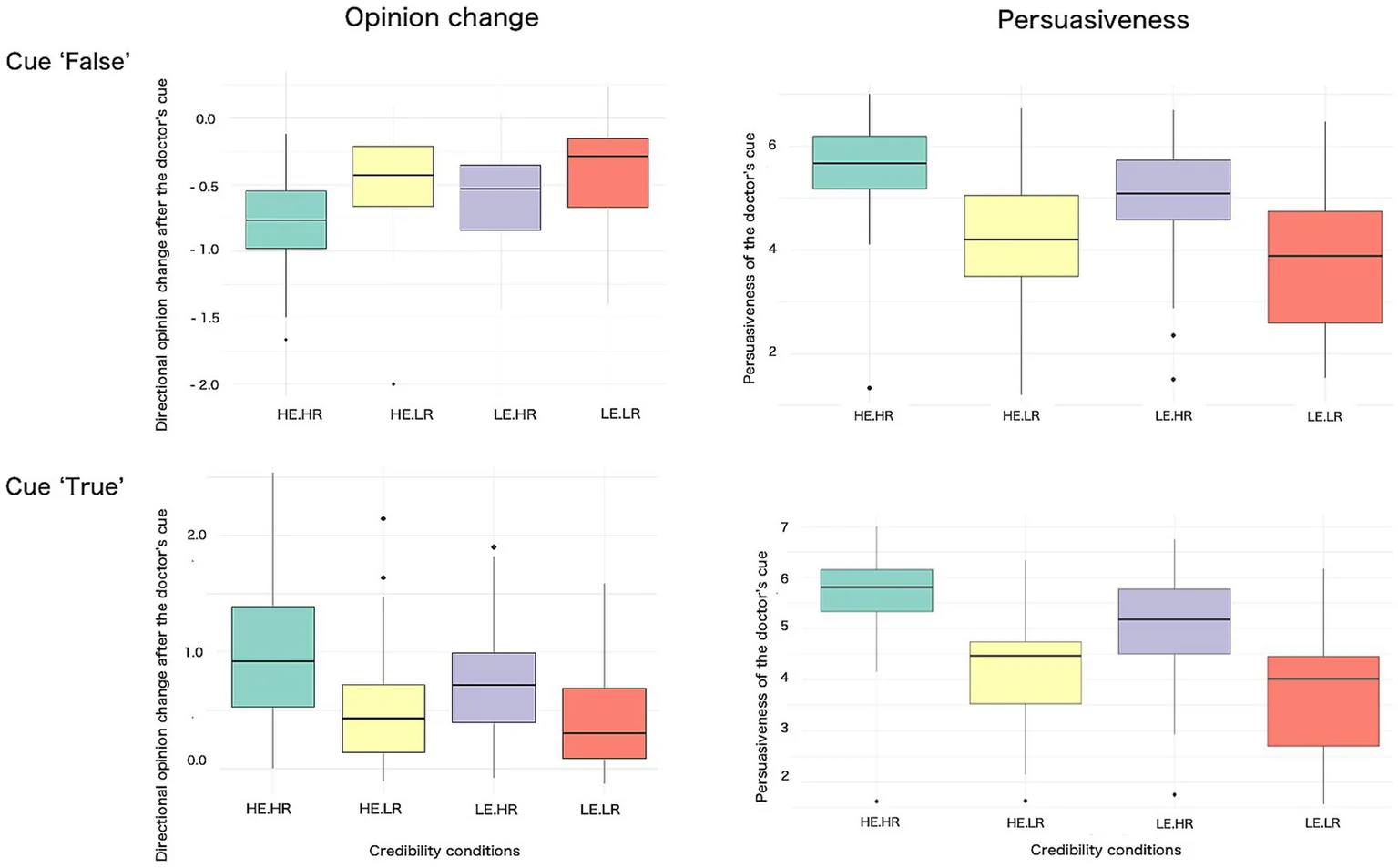
The descriptive panel of behavioral results of the persuasion task. Directional opinion change (left) and persuasiveness ratings (right) evoked by veracity cues from doctors across different levels of work experience and patient ratings. Data are presented separately for each veracity cue type (‘True’ and ‘False’). HE, High work experience; HR, High patient rating; LE, Low work experience; LR, Low patient rating.

#### Opinion changes

Linear mixed-effects models were used to examine the influence of doctor credibility (work experience, patient ratings), cue type (‘True’, ‘False’), and participants’ personality traits (CRT, NFCS, and CS scores) on both absolute and signed (directional) opinion change (see Table 2). Coefficients are reported in standardized form (outcome z-scored), so that *β* coefficients are expressed in standard-deviation units. Comparison of the random-effects structures favored the random-slopes specification, which was preferred over an intercepts-only model by AIC and by likelihood-ratio tests for all three outcomes (and by BIC for the signed opinion-change and persuasiveness models; see Supplementary Table S4); it is therefore reported throughout.

**Table 2:** Linear mixed-effects models examining the influence of credibility (work experience, patient rating), cue type (‘True’, ‘False’), and participants’ personality traits (CRT, NFCS, and CS scores) on absolute (left side) and signed (right side) opinion change.

Predictor	Absolute (|post − pre|) opinion change				Signed (post − pre) opinion change			
	*β*	SE	*t*	*p*	*β*	SE	*t*	*p*
Intercept	0.527	0.366	1.439	0.15	−0.877	0.148	−5.916	<0.001*
Cue (True)	0.208	0.110	1.885	0.059	1.577	0.139	11.376	<0.001*
Patient rating (low)	−0.285	0.082	−3.480	<0.001*	0.233	0.065	3.573	<0.001*
Work experience (low)	−0.188	0.078	−2.411	0.016*	0.152	0.062	2.445	0.015*
CRT	0.018	0.023	0.791	0.429	0.005	0.008	0.584	0.559
NFCS	−0.006	0.006	−1.045	0.296	0.002	0.002	0.782	0.434
CS	0.006	0.004	1.567	0.117	−0.002	0.001	−1.097	0.273
Cue × Patient rating	−0.179	0.107	−1.667	0.096	−0.611	0.117	−5.230	<0.001*
Cue × Work experience	−0.031	0.101	−0.312	0.755	−0.332	0.100	−3.325	<0.001*
Patient rating × Work experience	0.140	0.097	1.445	0.148	−0.114	0.076	−1.493	0.136
Cue × Patient rating × Work experience	0.027	0.132	0.205	0.838	0.251	0.117	2.141	0.032*

Random effects	Variance (σ^2^)	SD	Variance (σ^2^)	SD
Participant	0.235	0.485	0.172	0.414
Statement	0.074	0.272	0.050	0.223
Doctor profile	0.001	0.023	0.000	0.000
Residual	0.732	0.856	0.485	0.697

Significant values are marked by *. Models were fitted at the trial level with a fully crossed random-effects structure (random intercepts for participants, statements, and doctor profiles). In addition to these intercepts, the models include by-participant random slopes for cue, rating, experience and their interactions and a by-statement slope for cue, doctors profiles were modelled with a random intercept only; the full variance/SD components for all slope terms are reported in Supplementary Table S5. Reference levels: Cue = False, Rating = high, Experience = high.

The model for the absolute opinion change revealed significant main effects of both patient ratings (*β* = −0.285, SE = 0.082, *t* = −3.48, *p* < 0.001) and work experience (*β* = −0.188, SE = 0.078, *t* = −2.41, *p* = 0.016) on the magnitude of opinion change. A formal comparison of the two standardized effects indicated that they did not differ significantly (patient rating vs. work experience: χ^2^(1) = 1.72, *p* = 0.19). The main effect of cue type was only marginal (*β* = 0.208, SE = 0.110, *t* = 1.89, *p* = 0.059). No other factors or interactions reached significance.

Additionally, we refitted the same model using the signed (directional) measure of opinion change to test whether these effects reflect cue-consistent updating rather than undirected response variability. Relative to cues ‘False’, cues ‘True’ elicited a significant increase in agreement (*β* = 1.577, SE = 0.139, *t* = 11.376, *p* < 0.001), confirming that behavioral updating was aligned with the implication of the veracity cue.

Crucially, this cue-consistent updating was most strongly attenuated by patient ratings: the Cue (True) × Patient Rating (Low) interaction was large and highly significant (*β* = −0.611, SE = 0.117, *t* = −5.230, *p* < 0.001), indicating that a lower patient rating substantially reduced the cue-driven shift. Work experience moderated cue-consistent updating in the same direction but to a lesser extent [Cue (True) × Experience (Low): *β* = −0.332, SE = 0.100, *t* = −3.325, *p* < 0.001]. The standardized main effects of patient rating and work experience did not differ significantly in magnitude: χ^2^(1) = 1.91, *p* = 0.17. None of the personality traits significantly predicted signed opinion change (see Table 2, right side).

Examination of the random-effects structure confirmed that modeling these dependencies was warranted. Allowing the cue effect to vary across participants and statements substantially improved model fit over an intercepts-only structure for both measures (see Supplementary Table S4), reflecting considerable heterogeneity in how strongly individuals and individual statements were subject to cue-consistent updating: the by-participant random slope for cue was sizeable (signed σ^2^ = 0.557; absolute σ^2^ = 0.234), as was the by-statement cue slope (σ^2^ = 0.055 and 0.268, respectively; see Supplementary Table S5). Among the random intercepts, between-participant variance was moderate (absolute σ^2^ = 0.235; signed σ^2^ = 0.172) and statement-level variance smaller (σ^2^ = 0.074 and 0.050), whereas variation across doctor profiles was negligible (σ^2^ ≤ 0.001). The residual variance dominated overall (σ^2^ = 0.732 and 0.485 for the absolute and signed models, respectively), indicating that most variability arose at the level of individual trial responses rather than from stimulus- or profile-level clustering (full variance components in Supplementary Table S5).

#### Perceived persuasiveness of doctor’s veracity cues

A linear mixed-effects regression with random-slopes specification was conducted to examine whether doctor credibility (work experience, patient rating), participant personality traits (CRT, NFCS, CS), and cue type predicted persuasiveness ratings (see Table 3). Coefficients are reported in standardized form (outcome z-scored), so that *β* values are expressed in standard-deviation units. Both work experience (*β* = −0.352, SE = 0.117, *t* = −3.013, *p* = 0.003) and patient rating (*β* = −0.771, SE = 0.146, *t* = −5.281, *p* < 0.001) were significant predictors of perceived persuasiveness of doctor’s veracity cues. The negative coefficients indicate that low-credibility doctors were perceived as less persuasive, with patient ratings exerting a substantially stronger effect than work experience regarding the coefficients (*β* = −0.771 vs. *β* = −0.352). A formal comparison confirmed that this difference was statistically significant (patient rating vs. work experience: χ^2^(1) = 8.84, *p* = 0.003). Notably, and in contrast to the findings for opinion change, cue type did not significantly affect persuasiveness ratings (*β* = 0.045, SE = 0.071, *t* = 0.637, *p* = 0.524). None of the personality traits—CRT (*β* = 0.019, SE = 0.033, *t* = 0.580, *p* = 0.562), NFCS (*β* = −0.016, SE = 0.008, *t* = −1.906, *p* = 0.057) or CS (*β* = 0.003, SE = 0.006, *t* = 0.521, *p* = 0.603)—were significant predictors, and no interactions reached statistical significance.

**Table 3:** A linear mixed-effects model examining the influence of credibility (work experience, patient rating), cue type (‘True’, ‘False’), and participants’ personality traits (CRT, NFCS, and CS scores) on persuasiveness.

Predictor	*β*	SE	*t*	*p*
Intercept	1.512	0.515	2.934	0.003*
Cue (True)	0.045	0.071	0.637	0.524
Patient rating (low)	−0.771	0.146	−5.281	<0.001*
Work experience (low)	−0.352	0.117	−3.013	0.003*
CRT	0.019	0.033	0.580	0.562
NFCS	−0.016	0.008	−1.906	0.057
CS	0.003	0.006	0.521	0.603
Cue × Patient rating	−0.058	0.075	−0.765	0.444
Cue × Work experience	−0.016	0.070	−0.230	0.818
Rating × Work experience	0.118	0.135	0.879	0.379
Cue × Patient rating × Work experience	0.055	0.092	0.594	0.552

Random effects	Variance (σ^2^)	SD
Participant	0.263	0.513
Statement	0.021	0.146
Doctor profile	0.011	0.103
Residual	0.427	0.653

Significant values are marked by *. The model was fitted at the trial level with a fully crossed random-effects structure (random intercepts for participants, statements, and doctor profiles). In addition to these intercepts, the models include by-participant random slopes for cue, rating, experience and their interactions and a by-statement slope for cue, doctors profiles were modelled with a random intercept only; full slope variance/SD components are reported in Supplementary Table S5. Reference levels: Cue = False, Rating = high, Experience = high.

Examination of the random-effects structure indicated pronounced between-participant heterogeneity in how strongly individuals relied on source credibility when judging persuasiveness. This was most striking regarding sensitivity to the patient rating (by-participant rating slope σ^2^ = 0.505) and to work experience (σ^2^ = 0.231); baseline persuasibility also varied across participants (intercept σ^2^ = 0.263). This variation, echoing the opinion-change models, made the random-slopes structure strongly preferable to an intercepts-only specification (see Supplementary Table S4). Statement-level (σ^2^ = 0.021) and doctor-profile (σ^2^ = 0.011) intercept variances were comparatively small, whereas the residual variance accounted for a substantial portion of the total variation (σ^2^ = 0.427; see Supplementary Table S5 for complete variance components).

Overall, both work experience (expertise) and patient ratings significantly predicted absolute and signed opinion change and persuasiveness. Across all three outcomes the standardized effect of patient ratings was numerically larger than that of work experience, but this difference was significant only for persuasiveness. Thus, participants relied more on aggregate star ratings than on professional work experience specifically when judging persuasiveness. Need for cognition showed a non-significant negative trend in predicting persuasiveness ratings.

### EEG results: persuasion task (EEG session 1)

We focused the analysis on ERPs elicited by veracity cues (‘True’ and ‘False’) that were attributed to doctors with different levels of credibility. The permutation F-test demonstrated a significant difference in ERP to the veracity cue ‘False’ from doctors with high work experience/high patient ratings and low work experience/low patient rating doctors within the 158–214 ms time-window (mean *F* = 9.99, max *F* = 13.62, *p* = 0.02; see Figure 3). For the veracity cue ‘True’, no significant clusters were found in any time window.

**Figure 3:**
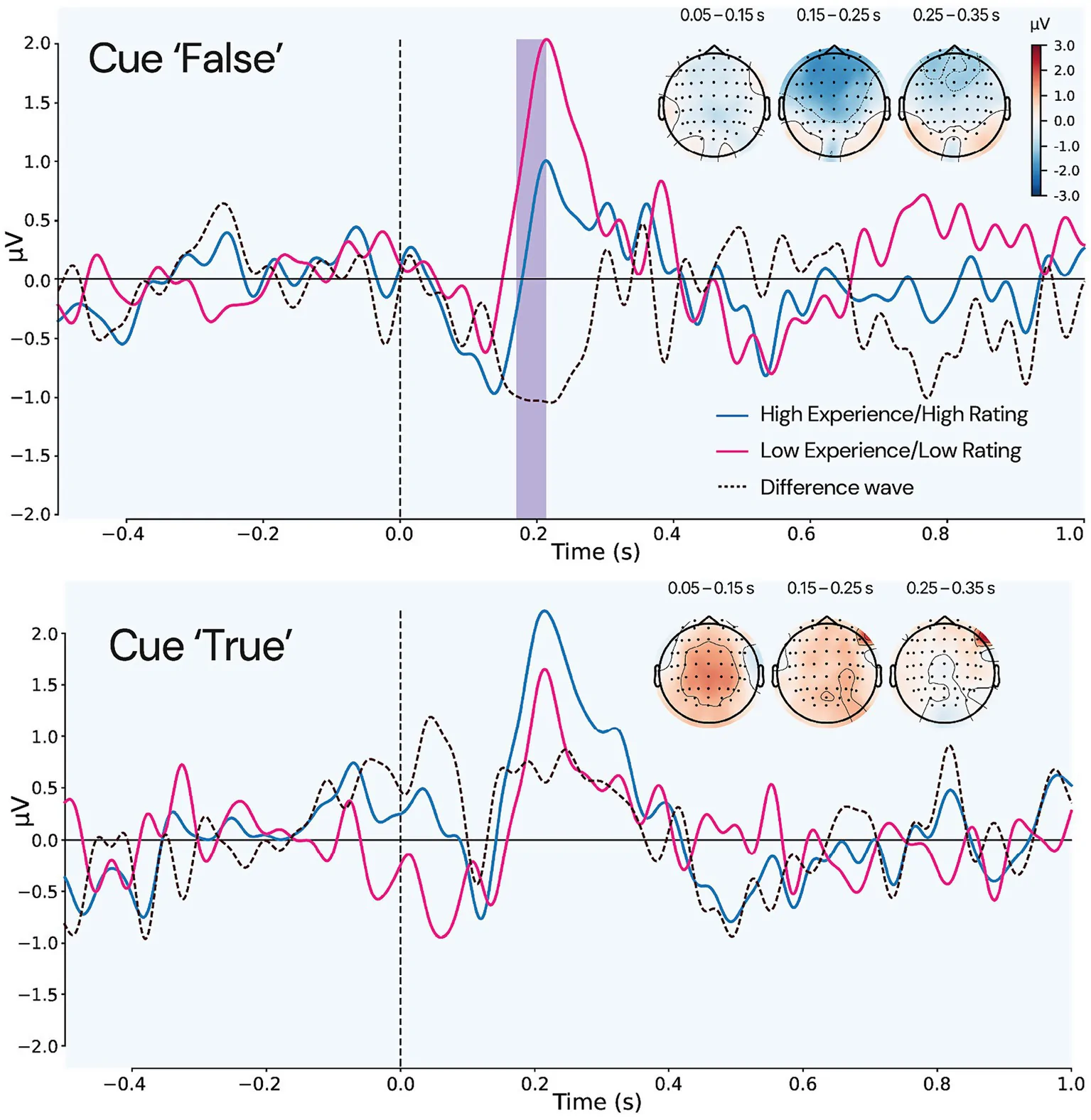
Grand-averaged ERPs for veracity cues ‘False’ and ‘True’ attributed to medical doctors with high work experience/high patient ratings versus low work experience/low patient ratings. The dashed line represents the difference waves, while the topographic maps illustrate the scalp distribution of these differences. The shaded region marks clusters of significant differences between ERPs.

To check whether the effect of credibility is driven primarily by the doctors’ patient ratings we further compared ERPs to veracity cues attributed to medical doctors with high work experience/high patient ratings and ones with high work experience/low patient ratings. The cluster-based permutation F-test also revealed a significant difference within the 162–204 ms time-window (mean *F* = 6.30, max *F* = 7.88, *p* = 0.047; see Figure 4). However, no significant clusters were observed for the veracity cue ‘True’ (all *p* > 0.05). No significant differences were found between ERPs in other experimental conditions (all *p* > 0.05; see Supplementary Figure S1–S3).

**Figure 4:**
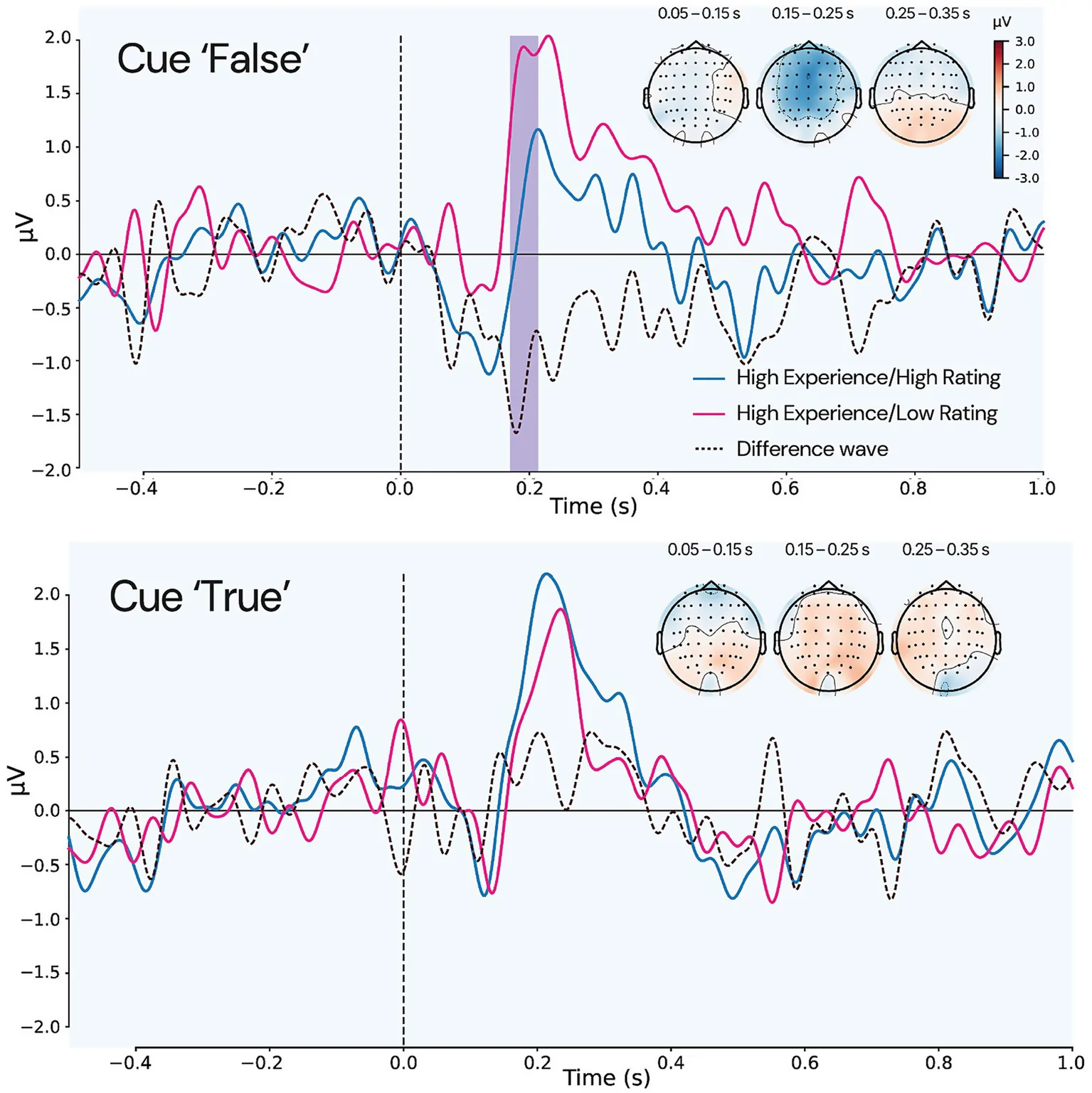
Grand-averaged ERPs for veracity cues ‘False’ and ‘True’ from medical doctors with high work experience/high patient ratings and high work experience/low patient ratings. The dashed line represents the difference waves, while the topographic maps illustrate the scalp distribution of these differences. The shaded region marks clusters of significant differences between ERPs.

Subsequent raw-scale linear regression analyses examining whether ERP amplitude on the approximate time window elicited from the permutation F-test (150–210 ms) predicted absolute and signed (directional) opinion change and persuasiveness yielded no significant effect of ERP amplitude (all *p* > 0.05; see Supplementary Tables S6–S8).

Additionally, we used sLORETA for source localization of the patient rating effect on the neural processing of the veracity cue ‘False’ (Pascual-Marqui et al., 2002; De Pascalis et al., 2010). Focusing on the approximate time window elicited from the permutation F-test (150–210 ms), we localized the brain sources of the difference wave between high experience/high patient ratings and high experience/low patient ratings conditions, thereby isolating the effect of patient ratings while controlling for experience level. Descriptive source analysis revealed activation in ventromedial frontal and posterior medial cortices (see Figure 5, left). A cluster-based permutation test at the source level confirmed a significant cluster in the posterior medial cortex (T ~ mean~ = 8.28, *p* = 0.042; see Figure 5, right), indicating that the ‘False’ veracity cue from highly rated doctors elicited significantly greater source activation in this region compared to doctors with low patient ratings.

**Figure 5:**
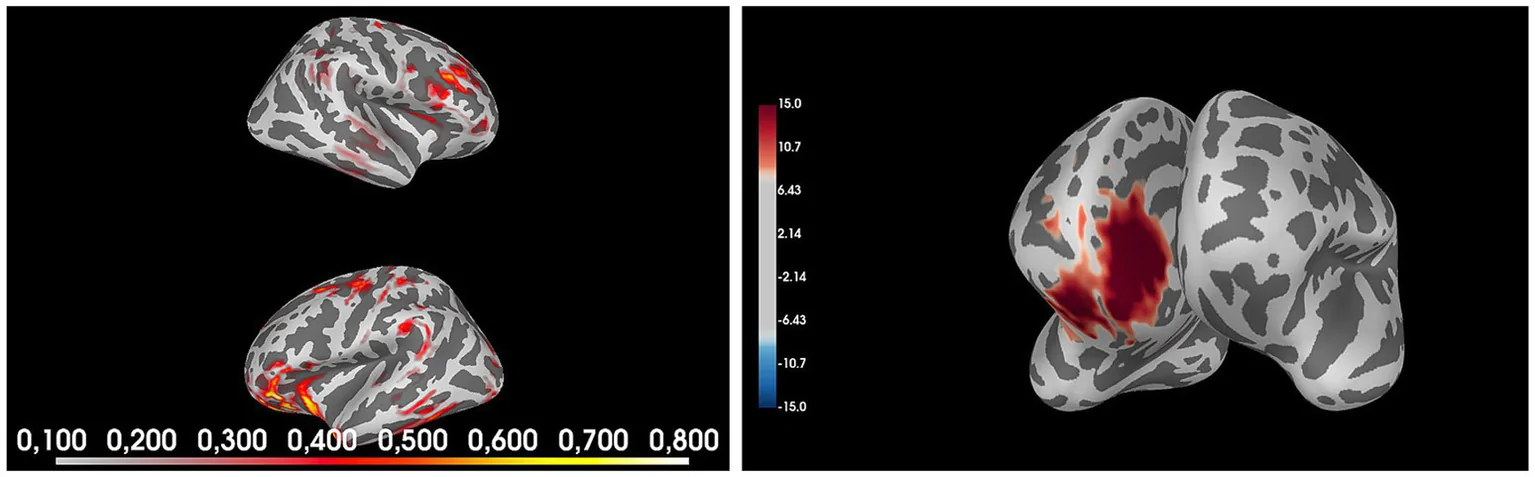
sLORETA descriptive source analysis (left) and a cluster-based permutation test (right) of the difference waves (approximate time window of 150–210 ms) for high experience/high patient ratings minus high experience/low patient ratings for cues ‘False’.

As a final confirmation, we conducted a three-way repeated-measures ANOVA with factors *Cue type* (cues ‘True’ vs. ‘False’), *Work experience* (high/low) and *Patient rating* (high/low) for ERP mean amplitudes in the traditional FRN(RewP) window (200–300 ms) taken from prior literature (e.g., Zhang et al., 2012; Zhang et al., 2017; Mei et al., 2018). We found a significant *Cue Type* × *Patient rating* interaction, indicating that specifically the veracity cue ‘False’ had the strongest effect when attributed to doctors with high patient ratings [*F*(1,35) = 5.77, *p* = 0.022; see Table 4].

**Table 4:** A three-way repeated-measures ANOVA for ERP amplitudes in the traditional FRN(RewP) window (200–300 ms).

Factor	*F*-value	*p*-value
Cue type	0.11	0.744
Work experience	0.05	0.819
Rating	0.02	0.896
Cue type × Work experience	1.97	0.170
Cue type × Patient rating	5.77	0.022*
Work experience × Patient rating	0.20	0.656
Cue type × Work experience × Patient rating	0.22	0.641

Significant values are marked by *.

### EEG results of the control EEG session 2

For the control study, permutation F-test revealed the significant difference of the grand-averaged ERPs to positive (smiley) and negative (frowny) feedback (F mean = 9.051, F max = 13.121, *p* = 0.047) at 194–262 ms time-window (see Supplementary Figure S4).

## Discussion

In an era of conflicting health advice, source credibility is one of the most important factors in determining how persuasive medical information is to patients. In the current study, participants were exposed to various medical headlines followed by veracity cues from doctors with different levels of credibility. The experimental design allowed us to separate the persuasive effects of the two source credibility cues: years of work experience and patient ratings (aggregate star ratings). It is important to note that both years of work experience and patient ratings function as composite indices of a doctor’s professional quality. However, in real-world online medical settings, years of work experience serve as a prominent marker of expertise. This is consistent with source-credibility research, which positions professional experience as a core constituent of perceived expertise (Ohanian, 1990; Guetz and Bidmon, 2023). Similarly, aggregated patient star ratings serve as a composite peripheral heuristic, simultaneously signaling inter-personal trust, institutional competence, and peer-endorsed social proof. Our behavioral results demonstrate that both high work experience and high patient ratings evoked greater opinion change and increased perceived persuasiveness of the veracity cues relative to their low-level counterparts. Interestingly, patient ratings exerted a significantly stronger behavioral effect than work experience on perceived persuasiveness, whereas for opinion change the two cues contributed comparably. In parallel, neurophysiological results showed that patient ratings of doctors’ (source) significantly modulated ERP responses to the veracity cues, but only for the ‘False’-cues condition. However, the ERP amplitude did not significantly predict opinion change or persuasiveness, leaving the link between this ERP modulation and subsequent belief updating an open question for future research.

Our behavioral results demonstrated that highly experienced and highly rated doctors elicited the most significant opinion change and the greatest perceived persuasiveness compared to doctors in other credibility conditions. Such findings are in line with early studies that suggest that the more credible the communication source, the greater the opinion change toward the advocated position (Hovland and Weiss, 1951; Kelman and Hovland, 1953) and further support the framework suggesting that the perceived credibility of a source serves as a primary heuristic that individuals use to judge a message’s persuasive strength, often bypassing more effortful argument evaluation (Pornpitakpan, 2004; Hovland and Weiss, 1951).

Regarding the existing debate on source credibility dimensions, our linear mixed-effects models revealed that patient ratings exerted a significantly stronger effect than doctors’ work experience on perceived persuasiveness. Because the ratings represent ecologically valid, yet multi-dimensional cues, we interpret this pattern as evidence that a rating-based, composite social signal can match, and in the case of persuasiveness surpass, the influence of an explicit marker of expertise.

In the medical context, where information is complex and outcomes are highly personal, participants likely relied on such peripheral cues as a heuristic to manage uncertainty and perceived risk (Ojha et al., 2025; Recchia et al., 2024). For example, a high rating might signal that the doctor prioritizes the patient’s best interests, which serves as a prerequisite for adhering to medical advice (Siegrist et al., 2000; Mayer et al., 1995). However, the apparent primacy of patient ratings over work experience should be interpreted with caution. Aggregated star ratings function as a compound peripheral cue that may simultaneously capture popularity, patient satisfaction, and perceived warmth, as well as perceived expertise (e.g., Hocevar et al., 2017; Metzger, 2007). Therefore, future studies are clearly needed to isolate the specific psychological constructs underlying the effect of patient ratings on the processing of medical veracity cues.

Finally, our linear mixed models revealed an asymmetry in how veracity cues influenced opinion change; specifically, ‘True’ cues from highly credible doctors elicited significantly stronger signed opinion change compared to ‘False’ cues. This pattern suggests that corrective (‘False’) feedback might encounter greater resistance at the behavioral level than confirmatory (‘True’) feedback (Edwards and Smith, 1996; Taber and Lodge, 2006).

Regarding the neurophysiological findings, source credibility significantly modulated the neural signatures of veracity cues ‘False’ within an early 150–210 ms time-window. More specifically, we observed a similar frontocentral ERP-correlate of source credibility when contrasting the high work experience/high patient ratings condition against both the low work experience/low patient ratings and the high work experience/low patient ratings conditions. These results suggest that veracity cues ‘False’ from doctors with high patient ratings selectively modulate early ERP amplitudes. Importantly, this modulation was also confirmed in the canonical 200–300 ms FRN(RewP) window (Zhang et al., 2012; Zhang et al., 2017; Mei et al., 2018), anchoring the effect within the standard FRN time range.

While the FRN(RewP) classically peaks around 250 ms, it has been operationalized across a broad latency range (Sambrook and Goslin, 2014), and an early-onset FRN(RewP)-like frontocentral negativity has been reported in social-evaluative paradigms (Shestakova et al., 2013). The FRN(RewP) component is increasingly recognized as reflecting expectation violations and surprise signals rather than purely reward prediction errors (RPE) (Alexander and Brown, 2011; Ferdinand et al., 2012; Hauser et al., 2014). Specifically regarding the RPE-FRN, a later measurement interval of 270–300 ms has been suggested to capture the strongest effects of outcome magnitude and likelihood. Previous EEG studies have consistently reported larger FRN amplitudes for unexpected compared to expected outcomes (Hajcak et al., 2007; Pfabigan et al., 2011) and for negative relative to positive performance feedback (Miltner et al., 1997; Nieuwenhuis et al., 2004). This principle extends to social contexts: van der Veen et al. (2016) showed that unexpected social judgments yielded larger FRN(RewP) than correctly predicted ones. Consequently, the FRN(RewP) is enhanced when outcomes contradict predictive beliefs (Glazer et al., 2018; Hajcak et al., 2007). This makes it highly relevant for the cognitive processing of veracity cues, where source characteristics shape the magnitude of expectancy violations.

Our findings can be interpreted within an expectation-based framework of source credibility processing. In our study, patient ratings might serve as a prior that shapes participants’ beliefs about veracity cue accuracy that modulated the salience of the corrective feedback (Bates et al., 2006; Metzger and Flanagin, 2013). The selective modulation of the FRN(RewP) by source credibility solely for ‘False’ cues aligns with the signed prediction error hypothesis, which posits that the component is primarily sensitive to outcomes that are worse than expected (Holroyd and Coles, 2002; Hajcak et al., 2007). Conversely, some evidence suggest that the FRN(RewP) reflects the motivational salience of outcomes (Pfabigan et al., 2011), consistent with the theory that dopaminergic systems encode surprise-like saliency signals (Redgrave and Gurney, 2006; Matsumoto and Hikosaka, 2009). Under this framework, the FRN(RewP) encodes prediction errors associated with salience rather than purely with motivational value; thus, it is typically stronger for negative feedback, as behavioral errors are often more motivationally salient than successes (Oliveira et al., 2007). Perhaps, work experience, as a more objective numerical indicator of expertise is processed systematically and exerts its influence through a more deliberate evaluation of veracity cues (Chaiken and Maheswaran, 1994). In contrast, patient ratings—representing an aggregated social signal of peer consensus—may function as a rapid heuristic that modulates earlier, automatic evaluative processing (Chaiken, 1980; Petty and Cacioppo, 1984; Sundar and Nass, 2001). Interestingly, some ERP evidence suggests that the early N100–P200 complex is sensitive to evaluation and decision-making processes regarding trust in news sources (Fan et al., 2021). This may explain why, in the current study, ‘True’ veracity cues did not show a statistically reliable credibility-modulated response, whereas frontocentral ERPs were highly sensitive to the source of ‘False’ cues. That said, the absence of a credibility effect for ‘True’ cues does not exclude a weaker, confirmation-related positivity (RewP) to ‘True’ signals from credible sources that the present design may have been underpowered to detect.

An important interpretive caveat applies to the differing perceptual formats of the two credibility cues: work experience was presented as a text-based number of years, whereas patient ratings appeared as a graphical star display. Because the star format is more visually salient, the tendency for the star rating dimension to modulate ERPs within the approximate 150–210 ms window may partly reflect this perceptual asymmetry, rather than an intrinsic difference in the neurocognitive processing of various source credibility cues. Importantly, however, the doctor’s profile card containing these metrics was no longer on screen during the presentation of the veracity cues—the precise period when the ERPs were recorded. Therefore, any effects of visual salience would have to operate indirectly, by top-down modulation of the subsequent processing of the veracity cues. Nonetheless, future studies should equalize the visual salience of information used to signal different credibility dimensions.

Our results indicate that, in contrast to confirmatory labels, ‘False’ veracity cues or corrective warnings trigger a source-dependent evaluative process at relatively early stages of cue processing. This early sensitivity is consistent with prior work showing that a frontocentral feedback negativity can arise early in the social-evaluative domain: disagreement with a normative group opinion elicits an FRN-like response at approximately 200 ms (Shestakova et al., 2013), and mediofrontal feedback signals track social rejection and social expectancy violation more generally (Sun and Yu, 2014; Schnuerch and Gibbons, 2015; Zubarev et al., 2017). Whether this early sensitivity to patient ratings is causally linked to subsequent belief revision remains an open empirical question, since our supplementary regression analyses did not reveal a direct association between these source-sensitive ERP responses and subsequent opinion change or persuasiveness. Nevertheless, the present effect converges with evidence that the amplitude of the mediofrontal feedback negativity is scaled by the salience and unexpectedness of evaluative feedback, rather than by reward value alone (Talmi et al., 2013; Pfabigan et al., 2011; Hauser et al., 2014), and that this salience effect is especially pronounced for socially corrective, expectancy-violating feedback, source-localized to the salience network (van der Molen et al., 2017). Within this body of work, our findings align with an interpretation where the FRN(RewP) reflects the salience of integrating corrective feedback weighted by aggregate star ratings. However, the present design cannot adjudicate between this account and alternative interpretations based on behavioral data alone.

Source localization further elucidated the neural substrates of credibility-modulated processing of veracity cues. For ‘False’ cues, the most robust neural response differentiating high and low credibility conditions was localized to frontal and posterior medial regions. This is consistent with the scalp topography of the FRN(RewP) and its proposed generators in the anterior cingulate and paracingulate cortex and medial frontal cortex (Holroyd and Coles, 2002; Miltner et al., 1997). Medial cortical regions play a central role in cognitive control, conflict, and error monitoring (Alexander and Brown, 2019). Such localization of the credibility-modulated ERPs aligns with the interpretation that source credibility amplifies the salience of the corrective feedback. Interestingly, the paracingulate cortex has been implicated in building trust relationships to predict others’ subsequent behavior (Krueger et al., 2007) and in social cognition (Amodio and Frith, 2006). On the other hand, the ventromedial frontal cortex plays a key role in a “valuation system” believed to be essential for value-based learning and decision-making (Bartra et al., 2013), while the frontal pole is involved in maintaining primary goals while processing sub-goals (Koechlin and Hyafil, 2007).

Importantly, the identity of this early medial-frontal component warrants careful consideration, as the FRN and RewP are not universally regarded as a single, independent process. Because the FRN and RewP represent two poles of the same difference wave, our findings could equally be interpreted through the RewP framework (Proudfit, 2015; Holroyd et al., 2008; Cavanagh and Holroyd, 2026), with the observed difference driven by a modulation of the positive evoked deflection. However, specificity of our observed effect to the negative veracity cue ‘False’ makes an FRN-centered interpretation quite plausible. Overlapping components may also contribute to observed credibility-related ERPs. Specifically, the frontocentral P2 (~170–230 ms) reflects stimulus-driven visual attention and structural feature selection (Crowley and Colrain, 2004), and it typically increases in response to incongruent (Freunberger et al., 2007), or threatening and negative stimuli (Kubota and Ito, 2007). For instance, an early frontocentral positivity was shown to distinguish true from false items within a comparable latency range during a rumor-veracity task (Ding et al., 2024). Concurrently, the subsequent N2 (~200–350 ms) reflects cognitive control, conflict monitoring, and task-relevant outcome evaluation (Folstein and Van Petten, 2008). The N2 exhibits enhanced negativity when a prepotent truthful response must be overridden—such as during deception (Fu et al., 2017; Wu et al., 2009)—and remains sensitive to the aversive evaluation of untrustworthy social attributes (Hein and Leue, 2021). In the context of our study, a credible doctor’s ‘False’ cue may elicit a larger P2 due to its increased salience and threat to prior beliefs, while simultaneously engaging N2-linked conflict monitoring and aversive evaluation as the credible source contradicts the participant’s original stance. We therefore regard the observed ERP effect as a member of the broader family of medial-frontal components whose exact identity our scalp-level design cannot fully isolate. Consequently, future work using source localization or component-decomposition approaches is needed to delineate its precise neural generators.

We acknowledge several limitations of this study. First, patient ratings were presented in a format of stars, which may be more visually salient than the text-based description of work experience. This difference in presentation could have differentially modulated attention to these two aspects of doctor credibility. However, the behavioral results demonstrated robust effects of work experience on both opinion change and persuasiveness, suggesting that visual layout alone cannot account for the differences in neurocognitive processing between these cues. Second, as previously discussed, patient ratings represent a complex social signal. Although our experimental instructions explicitly framed these star ratings as patients’ assessments of a doctor’s trustworthiness, the rating signal itself may have been processed as a compound credibility cue encompassing perceived reputation, satisfaction, and experiential endorsement, rather than as an isolated index of interpersonal trust. The behavioral and neural patterns observed here should therefore be understood as reflecting how participants integrate a multi-faceted cue relevant to both trust and social proof. Future work could further isolate the pure trust component of source credibility by decoupling it from adjacent social dimensions. Third limitation is the notable gender imbalance, which may introduce a systematic bias in credibility assessments. Importantly, the Wilcoxon rank-sum test revealed no significant impact of gender on opinion change (W = 1685.5, *p* = 0.61) or persuasiveness (W = 1831, *p* = 0.85). Future research should address these limitations by implementing more uniform visual formats for source credibility dimensions and recruiting gender-balanced samples.

## Conclusion

Both perceived work experience and patient ratings significantly modulated the behavioral effect of veracity cues (‘True’ and ‘False’) from medical doctors that confirmed or disconfirmed health-related headlines. Doctors with both high work experience and high patient ratings elicited the greatest opinion change and received the highest persuasiveness ratings relative to those with low work experience and low patient ratings. However, credibility-modulated ERPs were observed solely in response to veracity cues ‘False’. This frontocentral evoked response resembled an early FRN(RewP), elicited particularly by veracity cues from highly rated doctors. Overall, our findings suggest that source credibility modulates the effectiveness of correcting feedback for medical headlines and demonstrates the distinct neurocognitive mechanisms underlying the impact of credibility on confirmatory versus disconfirmatory veracity cues. Specifically, these results may imply that ‘True’ or confirmatory labels require less intensive neural processing, whereas ‘False’ or corrective warnings should be paired with high-credibility sources to maximize their cognitive impact and persuasive effectiveness.

## Data Availability

The datasets presented in this study can be found in online repositories. The names of the repository/repositories and accession number(s) can be found at: https://osf.io/6x7f9.
