# Supplementary material for: Neurocognitive mechanisms of source credibility: how work experience and patient ratings modulate the processing of medical veracity cues
Source: Front Hum Neurosci. 2026 Sep 18;20:1867901. doi: 10.3389/fnhum.2026.1867901 (PMC13630730; doi:10.3389/fnhum.2026.1867901)
Supplement: Supplementary file 1 [file Table_1.DOCX]

## **Table S1.** The medical headlines presented as stimuli material in the experiment

| Number | Veracity | Valence | Topic | Headline. Russian version | Headline. English version |
| --- | --- | --- | --- | --- | --- |
| 1 | TRUE | positive | vitamins | Здоровым детям не нужно принимать поливитамины. | Healthy children do not need to take multivitamins. |
| 2 | TRUE | negative | vitamins | Большие дозы витаминов могут быть токсичными. | Large doses of vitamins can be toxic. |
| 3 | FALSE | positive | vitamins | У витаминов нет срока годности. | Vitamins have no expiration date. |
| 4 | FALSE | negative | vitamins | Витамины, полученные из еды, лучше, чем витамины в таблетках. | Vitamins obtained from food are better than vitamins in tablets. |
| 5 | TRUE | positive | vitamins | Витамин K полезен для здоровья костей. | Vitamin K is beneficial for bone health. |
| 6 | TRUE | negative | vitamins | Прием витамина К может спровоцировать расстройство желудка. | Taking vitamin K can cause stomach upset. |
| 7 | FALSE | positive | vitamins | Чтобы получить норму витаминов группы К, достаточного включить зелень в свой рацион. | To get your daily requirement of vitamin K, it's enough to include greens in your diet. |
| 8 | FALSE | negative | vitamins | Укол витамина К способен вызвать лейкемию у ребенка. | A vitamin K injection can cause leukemia in a child. |
| 9 | TRUE | positive | lifestyle | Глазирователи, которыми смазывают фрукты перед продажей, безвредны для здоровья. | Glazing agents used to coat fruits before sale are harmless to health. |
| 10 | TRUE | negative | lifestyle | Ранние арбузы могут содержать нитраты, опасные для здоровья. | Early watermelons may contain nitrates that are dangerous to health. |
| 11 | FALSE | positive | lifestyle | Бананы не нужно мыть перед употреблением в пищу. | Bananas don't need to be washed before eating. |
| 12 | FALSE | negative | lifestyle | Чтобы фрукты дольше хранились, их смазывают опасными для здоровья химическими составами. | To make fruits last longer, they are coated with chemical compounds dangerous to health. |
| 13 | TRUE | positive | lifestyle | Овощи достаточно тщательно промыть в проточной чистой воде. | Vegetables just need to be thoroughly rinsed in clean running water. |
| 14 | TRUE | negative | lifestyle | Фрукты, которые долго хранились, менее витаминные, чем сезонные. | Fruits that have been stored for a long time are less vitamin-rich than seasonal ones. |
| 15 | FALSE | positive | lifestyle | Сухофрукты не нужно мыть перед употреблением в пищу. | Dried fruits don't need to be washed before eating. |
| 16 | FALSE | negative | lifestyle | Чтобы смыть химикаты с поверхности фруктов, их нужно мыть в горячей воде с моющими средствами. | To wash off chemicals from fruit surfaces, they need to be washed in hot water with detergents. |
| 17 | TRUE | positive | lifestyle | Отказ от курения снижает уровень холестерина. | Quitting smoking reduces cholesterol levels. |
| 18 | TRUE | negative | lifestyle | Высокий уровень холестерина обычно не имеет симптомов. | High cholesterol usually has no symptoms. |
| 19 | FALSE | positive | lifestyle | Высокий уровень холестерина обычно сопровождается явными симптомами. | High cholesterol is usually accompanied by obvious symptoms. |
| 20 | FALSE | negative | lifestyle | Любой холестерин вреден. | Any cholesterol is harmful. |
| 21 | TRUE | positive | lifestyle | Здоровый образ жизни способствует поддержанию нормального уровня холестерина. | A healthy lifestyle helps maintain normal cholesterol levels. |
| 22 | TRUE | negative | lifestyle | Даже при нормальном весе уровень холестерина может быть высоким. | Even with normal weight, cholesterol levels can be high. |
| 23 | FALSE | positive | lifestyle | Людям до 40 лет не нужно проверять уровень холестерина. | People under 40 don't need to check their cholesterol levels. |
| 24 | FALSE | negative | lifestyle | Нужно избегать продуктов, содержащих холестерин. | Products containing cholesterol should be avoided. |
| 25 | TRUE | positive | lifestyle | Сахарозаменители снижают риск возникновения кариеса. | Sugar substitutes reduce the risk of cavities. |
| 26 | TRUE | negative | lifestyle | Некоторые виды сахарозаменителей повышают риск развития рака. | Some types of sugar substitutes increase the risk of cancer. |
| 27 | FALSE | positive | lifestyle | Сахарозаменитель можно использовать как лекарство для похудения. | Sugar substitutes can be used as a weight loss medication. |
| 28 | FALSE | negative | lifestyle | Сахарозаменители вызывают диабет. | Sugar substitutes cause diabetes. |
| 29 | TRUE | positive | *vaccine* | Серьезные побочные эффекты вакцин чрезвычайно редки. | Serious side effects from vaccines are extremely rare. |
| 30 | TRUE | negative | *vaccine* | Редкие болезни появятся вновь, если не прививать население. | Rare diseases will reappear if the population is not vaccinated. |
| 31 | FALSE | positive | *vaccine* | В вакцинах не будет необходимости, когда вырастет уровень санитарии и гигиены. | Vaccines won't be necessary when sanitation and hygiene levels improve. |
| 32 | FALSE | negative | *vaccine* | Большинство вакцин вызывают ряд опасных побочных эффектов, которые еще не известны. | Most vaccines cause a number of dangerous side effects that are not yet known. |
| 33 | TRUE | positive | *vaccine* | Одновременное введение нескольких вакцин безопасно для иммунной системы ребенка. | Simultaneous administration of multiple vaccines is safe for a child's immune system. |
| 34 | TRUE | negative | *vaccine* | Возбудители инфекций могут пересекать границы государств. | Disease pathogens can cross state borders. |
| 35 | FALSE | positive | *vaccine* | Если нет пандемий, то нет смысла прививаться от болезней. | If there are no pandemics, there is no point in getting vaccinated against diseases. |
| 36 | FALSE | negative | *vaccine* | Одновременное введение ребенку более одной вакцины пагубно влияет на его иммунную систему | Simultaneous administration of more than one vaccine to a child has a detrimental effect on their immune system. |
| 37 | TRUE | positive | *vaccine* | В одной дозе вакцины ртути меньше, чем городской ребенок принимает с пищей, водой и воздухом в течение дня | One dose of vaccine contains less mercury than an urban child receives from food, water and air during a day. |
| 38 | TRUE | negative | *vaccine* | Многие инфекции могут распространяться независимо от степени нашей чистоплотности | Many infections can spread regardless of our level of cleanliness. |
| 39 | FALSE | positive | *vaccine* | Лучше получить иммунитет в результате болезни, чем вакцинации. | It is better to gain immunity as a result of illness than vaccination. |
| 40 | FALSE | negative | *vaccine* | Вакцины содержат опасные для здоровья соединения, например, ртуть. | Vaccines contain compounds dangerous to health, such as mercury. |
| 41 | TRUE | positive | *vaccine* | Вакцина против кори-паротита-краснухи не вызывает аутизм | The measles-mumps-rubella vaccine does not cause autism. |
| 42 | TRUE | negative | *vaccine* | Универсальной вакцины от гриппа не существует | A universal flu vaccine does not exist. |
| 43 | FALSE | positive | *vaccine* | Некоторые вакцины являются надежной профилактикой аутизма | Some vaccines are reliable prevention of autism. |
| 44 | FALSE | negative | *vaccine* | Вакцины приводят к развитию у детей аутизма | Vaccines lead to the development of autism in children. |
| 45 | TRUE | positive | *vaccine* | Вакцинирование помогает избежать серьезных осложнений, вызываемых инфекционным заболеванием | Vaccination helps avoid serious complications caused by infectious diseases. |
| 46 | TRUE | negative | *vaccine* | Вакцины могут вызывать аллергическую реакцию у некоторых людей | Vaccines can cause allergic reactions in some people. |
| 47 | FALSE | positive | *vaccine* | Все вакцины являются гипоалергенными | All vaccines are hypoallergenic. |
| 48 | FALSE | negative | *vaccine* | Использование любой вакцины приводит к аллергическим реакциям | Using any vaccine leads to allergic reactions. |
| 49 | TRUE | positive | vitamins | Витамин Д снижает риск развития некоторых видов рака. | Vitamin D reduces the risk of developing certain types of cancer. |
| 50 | TRUE | negative | vitamins | Чрезмерное потребление витамина D приводит к отложению солей кальция в почках. | Excessive consumption of vitamin D leads to calcium salt deposits in the kidneys. |
| 51 | FALSE | positive | vitamins | Витамин D снижает артериальное давление. | Vitamin D lowers blood pressure. |
| 52 | FALSE | negative | vitamins | Употребление витамина D усугубляет депрессию. | Consuming vitamin D worsens depression. |
| 53 | TRUE | positive | altMedicine | Регулярное употребление отвара шиповника может снизить риск сердечно-сосудистых заболеваний. | Regular consumption of rosehip decoction can reduce the risk of cardiovascular diseases. |
| 54 | TRUE | negative | altMedicine | Чрезмерное употребление отвара шиповника может навредить слизистой оболочке желудка | Excessive consumption of rosehip decoction can harm the gastric mucosa. |
| 55 | FALSE | positive | altMedicine | Отвар шиповника помогает в работе печени | Rosehip decoction helps liver function. |
| 56 | FALSE | negative | altMedicine | Отвар шиповника повышает уровень сахара в крови | Rosehip decoction increases blood sugar levels. |
| 57 | TRUE | positive | altMedicine | Употребление чеснока профилактирует раковые заболевания. | Consuming garlic prevents cancer. |
| 58 | TRUE | negative | altMedicine | Чрезмерное употребление чеснока может спровоцировать изжогу. | Excessive consumption of garlic can trigger heartburn. |
| 59 | FALSE | positive | altMedicine | Чеснок - эффективное средство укрепления иммунитета. | Garlic is an effective means of strengthening immunity. |
| 60 | FALSE | negative | altMedicine | Настой чеснока вызывает болезни сердца | Garlic infusion causes heart disease. |
| 61 | TRUE | positive | lifestyle | Обработка фруктов соком алоэ увеличивет срок их хранения. | Treating fruits with aloe juice increases their shelf life. |
| 62 | TRUE | negative | lifestyle | Чрезмерное употребление алоэ может вызвать проблемы с пищеварительной системой. | Excessive consumption of aloe can cause digestive system problems. |
| 63 | FALSE | positive | lifestyle | Алоэ помогает вылечиться от коронавируса | Aloe helps cure coronavirus. |
| 64 | FALSE | negative | lifestyle | Употребление алоэ может спровоцировать рак пищевода | Consuming aloe can trigger esophageal cancer. |
| 65 | TRUE | positive | altMedicine | Регулярное употребление картофеля снижает уровень артериального давления. | Regular consumption of potatoes lowers blood pressure. |
| 66 | TRUE | negative | altMedicine | Употребление картофеля с ростками на клубне опасно для организма. | Consuming potatoes with sprouts on the tuber is dangerous to the body |
| 67 | FALSE | positive | altMedicine | Зеленоватый картофель можно употреблять в пищу | Greenish potatoes can be consumed |
| 68 | FALSE | negative | altMedicine | Картофель вреден для пищеварительной системы | Potatoes are harmful to the digestive system |
| 69 | TRUE | positive | lifestyle | Занятия йогой снижают уровень холестерина | Practicing yoga lowers cholesterol levels |
| 70 | TRUE | negative | lifestyle | Занятия йогой травмоопасны | Practicing yoga is prone to injuries |
| 71 | FALSE | positive | lifestyle | Занятия йогой помогают при лечении функциональных желудочно-кишечных расстройств | Practicing yoga helps in treating functional gastrointestinal disorders |
| 72 | FALSE | negative | lifestyle | Занятия йогой вредна для суставов | Practicing yoga is harmful to joints |
| 73 | TRUE | positive | lifestyle | Веганская диета профилактирует сердечно-сосудистые заболевания. | A vegan diet prevents cardiovascular diseases |
| 74 | TRUE | negative | lifestyle | Веганская диета негативно сказывается на прочности костной массы. | A vegan diet negatively affects bone strength |
| 75 | FALSE | positive | lifestyle | Рацион сыроеда способен обеспечить организм всеми необходимыми микроэлементами | A raw food diet can provide the body with all necessary microelements |
| 76 | FALSE | negative | lifestyle | Веганская диета повышает риск развития сердечно-сосудисых заболеваний. | A vegan diet increases the risk of developing cardiovascular diseases |
| 77 | TRUE | positive | lifestyle | Курение помогает справиться с тревожностью | Smoking helps cope with anxiety |
| 78 | TRUE | negative | lifestyle | Пассивное курение увеличивает шанс развития рака легких | Passive smoking increases the chance of developing lung cancer |
| 79 | FALSE | positive | lifestyle | Электронные сиграеты не вызывают привыкания к никотину | Electronic cigarettes do not cause nicotine addiction |
| 80 | FALSE | negative | lifestyle | Курение не опасно при беременности | Smoking is not dangerous during pregnancy |
| 81 | TRUE | positive | altMedicine | Контрастный душ помогает восстановлению мышц после физических нагрузок | Contrast showers help muscle recovery after physical exertion |
| 82 | TRUE | negative | altMedicine | Долгое принятие контрастного душа вызывает мышечное утомление | Long-term contrast showers cause muscle fatigue |
| 83 | FALSE | positive | altMedicine | Контрастный душ ускоряет выздоровление при вирусных заболеваниях | Contrast showers speed up recovery from viral diseases |
| 84 | FALSE | negative | altMedicine | Контрастный душ ослабляет иммунитет | Contrast showers weaken immunity |
| 85 | TRUE | positive | gmo | Продукты с ГМО безопасны для здоровья человека | GMO products are safe for human health |
| 86 | TRUE | negative | gmo | Генно-модифицированные растения быстрее истощают почву | Genetically modified plants deplete soil faster |
| 87 | FALSE | positive | gmo | Продукты с ГМО не оставляют никакого следа в организме человека | GMO products leave no trace in the human body |
| 88 | FALSE | negative | gmo | Генно-модифицированная трава убивает скот | Genetically modified grass kills livestock |
| 89 | TRUE | positive | gmo | Продукты с ГМО не изменяют ДНК человека | GMO products do not alter human DNA |
| 90 | TRUE | negative | gmo | Продукты с ГМО содержат несвойственные для них ДНК | GMO products contain DNA not characteristic of them |
| 91 | FALSE | positive | gmo | Все продукты с ГМО полезны для организма | All GMO products are beneficial to the body |
| 92 | FALSE | negative | gmo | Продукты с ГМО опасны для здоровья | GMO products are dangerous to health |
| 93 | TRUE | positive | gmo | Генная модификация может придавать растениям полезные свойства, например, устойчивость к вредителям | Genetic modification can give plants useful properties, such as pest resistance |
| 94 | TRUE | negative | gmo | Продукты с ГМО теряют во вкусовых качествах по сравнению с традиционными продуктами | GMO products lose taste qualities compared to traditional products |
| 95 | FALSE | positive | gmo | Все продукты на основе ГМО проходят тщательную проверку | All GMO-based products undergo thorough testing |
| 96 | FALSE | negative | gmo | Продукты с ГМО вызывают мутации | GMO products cause mutations |
| 97 | TRUE | positive | gmo | В России существует эффективная система санитарного контроля за ГМО-продукцией. | Russia has an effective system of sanitary control over GMO products |
| 98 | TRUE | negative | gmo | Ежегодно в России выявляются незарегистрированные линии ГМО. | Unregistered GMO lines are identified annually in Russia |
| 99 | FALSE | positive | gmo | Большинство ГМО-лекарственных препаратов эффективнее традицинонных. | Most GMO pharmaceutical drugs are more effective than traditional ones |
| 100 | FALSE | negative | gmo | Продукты с ГМО приводят к бесплодию. | GMO products lead to infertility |
| 101 | TRUE | positive | lifestyle | Здоровый сон помогает поддерживать хорошее психоэмоциональное состояние | Healthy sleep helps maintain good psychological and emotional state |
| 102 | TRUE | negative | lifestyle | Длительное недосыпание может вызвать бессоницу | Prolonged sleep deprivation can cause insomnia |
| 103 | FALSE | positive | lifestyle | Время суток не имеет значения для качества сна | Time of day does not matter for sleep quality |
| 104 | FALSE | negative | lifestyle | Длительный сон негативно сказывается на психоэмоциональном состоянии | Prolonged sleep negatively affects psychological and emotional state |
| 105 | TRUE | positive | lifestyle | Пища с глютеном это источник пробиотиков | Food with gluten is a source of probiotics |
| 106 | TRUE | negative | lifestyle | Непереносимость глютена ведет к нарушению пищеварения | Gluten intolerance leads to digestive disorders |
| 107 | FALSE | positive | lifestyle | Исключение глютеносодержащих продуктов благотворно влияет на пищеварительную систему | Excluding gluten-containing products has a beneficial effect on the digestive system |
| 108 | FALSE | negative | lifestyle | Глютен является одной из причин набора лишнего веса | Gluten is one of the causes of excess weight gain |
| 109 | TRUE | positive | lifestyle | Регулярное употребление протеина ускоряет рост мышечной массы у спортсменов | Regular protein consumption accelerates muscle mass growth in athletes |
| 110 | TRUE | negative | lifestyle | Некоторые добавки в смесях для протеиновых коктейлей несут вред для организма | Some additives in mixes for protein shakes are harmful to the body |
| 111 | FALSE | positive | lifestyle | Протеиновые коктейли содержат не только белок, но и множество других полезных элементов | Protein shakes contain not only protein but also many other beneficial elements |
| 112 | FALSE | negative | lifestyle | Спортивный протеин содержит искусственный белок (не натуральный) | Sports protein contains artificial protein (not natural) |
| 113 | TRUE | positive | lifestyle | 3% раствор перекиси водорода оказывает временное антисептическое действие | 3% hydrogen peroxide solution provides temporary antiseptic action |
| 114 | TRUE | negative | lifestyle | 3% раствор перекиси водорода недостаточен, чтобы убить все бактерии в ране | 3% hydrogen peroxide solution is insufficient to kill all bacteria in a wound |
| 115 | FALSE | positive | lifestyle | 3% раствор перекиси водорода способен обеззараживать любую рану | 3% hydrogen peroxide solution can disinfect any wound |
| 116 | FALSE | negative | lifestyle | Передозировка 3% раствором перекиси водорода усугубляет воспалительные процессы | Overdose of 3% hydrogen peroxide solution worsens inflammatory processes |
| 117 | TRUE | positive | altMedicine | Манту можно мочить | Mantoux test can be wetted |
| 118 | TRUE | negative | altMedicine | Туберкулез может поражать не только легкие, но и другие ткани организма | Tuberculosis can affect not only the lungs but also other body tissues |
| 119 | FALSE | positive | altMedicine | Туберкулез может пройти сам собой | Tuberculosis can go away on its own |
| 120 | FALSE | negative | altMedicine | Собачье мясо лечит туберкулез | Dog meat cures tuberculosis |
