# Supplementary material for: Neurocognitive mechanisms of source credibility: how work experience and patient ratings modulate the processing of medical veracity cues
Source: Front Hum Neurosci. 2026 Sep 18;20:1867901. doi: 10.3389/fnhum.2026.1867901 (PMC13630730; doi:10.3389/fnhum.2026.1867901)
Supplement: Supplementary file 2 [file Table_2.DOCX]

## **Table S2.** Friedman tests for opinion change and perceived persuasiveness across the four credibility conditions (high/low work experience × high/low patient rating).

| Variable | Analysis | χ² | df | p |
| --- | --- | --- | --- | --- |
| Opinion change | Collapsed across cue | 28.874 | 3 | < 0.001 |
| Opinion change | Cue 'True' | 30.277 | 3 | < 0.001 |
| Opinion change | Cue 'False' | 6.380 | 3 | 0.09 |
| Persuasiveness | Collapsed across cue | 64.633 | 3 | < 0.001 |
| Persuasiveness | Cue 'True' | 70.233 | 3 | < 0.001 |
| Persuasiveness | Cue 'False' | 48.6 | 3 | < 0.001 |
