# Supplementary material for: Neurocognitive mechanisms of source credibility: how work experience and patient ratings modulate the processing of medical veracity cues
Source: Front Hum Neurosci. 2026 Sep 18;20:1867901. doi: 10.3389/fnhum.2026.1867901 (PMC13630730; doi:10.3389/fnhum.2026.1867901)
Supplement: Supplementary file 3 [file Table_3.DOCX]

## **Table S3.** Bonferroni-corrected pairwise comparisons for opinion change and perceived persuasiveness across credibility conditions.

| Variable | Analysis | Comparison | Statistic | p-adj |
| --- | --- | --- | --- | --- |
| Opinion change | Collapsed | HE/HR vs. LE/LR | t(35) = 5.673 | < 0.001 |
| Opinion change | Collapsed | LE/HR vs. LE/LR | t(35) = 4.328 | < 0.001 |
| Opinion change | Collapsed | HE/HR vs. LE/HR | t(35) = 2.903 | 0.016 |
| Opinion change | Collapsed | HE/HR vs. HE/LR | V = 546 | < 0.001 |
| Opinion change | Collapsed | HE/LR vs. LE/LR | V = 423 | 0.403 |
| Opinion change | Cue 'True' | HE/HR vs. LE/LR | V = 624 | < 0.001 |
| Opinion change | Cue 'True' | HE/HR vs. HE/LR | V = 579 | < 0.001 |
| Opinion change | Cue 'True' | HE/HR vs. LE/HR | t(35) = 2.472 | 0.046 |
| Opinion change | Cue 'True' | LE/HR vs. LE/LR | V = 570 | < 0.001 |
| Opinion change | Cue 'True' | HE/LR vs. LE/LR | V = 402 | 0.188 |
| Opinion change | Cue 'False' | HE/HR vs. LE/LR | V = 497.5 | 0.007 |
| Opinion change | Cue 'False' | HE/HR vs. HE/LR | V = 498.5 | 0.023 |
| Opinion change | Cue 'False' | HE/HR vs. LE/HR | V = 416 | 0.097 |
| Opinion change | Cue 'False' | LE/HR vs. LE/LR | t(35) = 1.856 | 0.036 |
| Opinion change | Cue 'False' | HE/LR vs. LE/LR | V = 356 | 0.361 |
| Persuasiveness | Collapsed | HE/HR vs. LE/LR | V = 663 | < 0.001 |
| Persuasiveness | Collapsed | LE/HR vs. LE/LR | V = 646 | < 0.001 |
| Persuasiveness | Collapsed | HE/HR vs. HE/LR | V = 650 | < 0.001 |
| Persuasiveness | Collapsed | HE/HR vs. LE/HR | V = 598 | < 0.001 |
| Persuasiveness | Collapsed | HE/LR vs. LE/LR | t(35) = 3.983 | < 0.001 |
| Persuasiveness | Cue 'True' | HE/HR vs. LE/LR | V = 666 | < 0.001 |
| Persuasiveness | Cue 'True' | HE/HR vs. HE/LR | V = 653 | < 0.001 |
| Persuasiveness | Cue 'True' | HE/HR vs. LE/HR | V = 594.5 | < 0.001 |
| Persuasiveness | Cue 'True' | LE/HR vs. LE/LR | t(35) = 7.030 | < 0.001 |
| Persuasiveness | Cue 'True' | HE/LR vs. LE/LR | t(35) = 3.817 | 0.001 |
| Persuasiveness | Cue 'False' | HE/HR vs. LE/LR | V = 643 | < 0.001 |
| Persuasiveness | Cue 'False' | HE/HR vs. HE/LR | V = 629 | < 0.001 |
| Persuasiveness | Cue 'False' | HE/HR vs. LE/HR | V = 554 | < 0.001 |
| Persuasiveness | Cue 'False' | LE/HR vs. LE/LR | V = 618 | < 0.001 |
| Persuasiveness | Cue 'False' | HE/LR vs. LE/LR | t(35) = 2.951 | 0.014 |

*Note.* For opinion change to cue 'False', the omnibus Friedman test was non-significant (Table S2); the two comparisons listed are exploratory, and all remaining pairwise comparisons were non-significant (p-adj > 0.05). Paired t-tests (df = 35) are reported for normally distributed comparisons; Wilcoxon signed-rank tests (V) otherwise. p-adj = Bonferroni-adjusted p-value. HE – high work experience, HR – high patient rating, LE – low work experience, LR – low patient rating.
