# Supplementary material for: Neurocognitive mechanisms of source credibility: how work experience and patient ratings modulate the processing of medical veracity cues
Source: Front Hum Neurosci. 2026 Sep 18;20:1867901. doi: 10.3389/fnhum.2026.1867901 (PMC13630730; doi:10.3389/fnhum.2026.1867901)
Supplement: Supplementary file 4 [file Table_4.DOCX]

## **Table S4.** Comparison of random-effects structures for absolute opinion change, signed opinion change and persuasiveness (AIC/BIC and LRT).

| **Outcome** | Model | AIC | BIC | logLik | LRT (complex vs simple) |
| --- | --- | --- | --- | --- | --- |
| **Signed opinion change** | Simple | 8715.0 | 8808.2 | −4342.5 | — |
|  | Complex | 8263.2 | 8586.1 | −4079.6 | χ²(37) = 525.84, p < .001 |
| **Absolute opinion change** | Simple | 9831.7 | 9924.9 | −4900.9 | — |
|  | Complex | 9773.4 | 10096.3 | −4834.7 | χ²(37) = 132.3  , p < .001 |
| **Persuasiveness** | Simple | 8651.1 | 8744.3 | –4310.6 | — |
|  | Complex | 8008.6 | 8331.5 | −3952.3 | χ²(37) = 716.6, p < .001 |

*Note.* N = 3676 (120 statements, 36 participants, 16 doctor profiles). "Simple" = random intercepts for participants, statements, and doctor profiles. "Complex" = additionally by-participant slopes for cue, rating, experience and their interactions and a by-statement slope for cue. Likelihood-ratio tests (LRT) compare complex vs simple (df = 37). Lower AIC/BIC indicate better fit.
