# Supplementary material for: Neurocognitive mechanisms of source credibility: how work experience and patient ratings modulate the processing of medical veracity cues
Source: Front Hum Neurosci. 2026 Sep 18;20:1867901. doi: 10.3389/fnhum.2026.1867901 (PMC13630730; doi:10.3389/fnhum.2026.1867901)
Supplement: Supplementary file 5 [file Table_5.DOCX]

## **Table S5.** Variance (and standard deviation) of every random-effects term in the random-slopes (complex) models, for absolute and signed opinion change and persuasiveness.

| **Random effect** | **Abs Var** | **Abs SD** | **Signed Var** | **Signed SD** | **Persu Var** | **Persu SD** |
| --- | --- | --- | --- | --- | --- | --- |
| Statement: Intercept | 0.074 | 0.272 | 0.050 | 0.223 | 0.021 | 0.146 |
| Statement: Cue [True] | 0.268 | 0.518 | 0.055 | 0.234 | 0.135 | 0.367 |
| Participant: Intercept | 0.235 | 0.485 | 0.172 | 0.414 | 0.263 | 0.513 |
| Participant: Cue [True] | 0.234 | 0.484 | 0.557 | 0.747 | 0.040 | 0.201 |
| Participant: Experience [low] | 0.092 | 0.304 | 0.062 | 0.249 | 0.231 | 0.481 |
| Participant: Rating [low] | 0.112 | 0.335 | 0.074 | 0.272 | 0.505 | 0.711 |
| Participant: Cue × Experience | 0.126 | 0.355 | 0.199 | 0.446 | 0.037 | 0.193 |
| Participant: Cue × Rating | 0.172 | 0.415 | 0.331 | 0.576 | 0.061 | 0.248 |
| Participant: Experience × Rating | 0.081 | 0.284 | 0.053 | 0.231 | 0.128 | 0.358 |
| Participant: Cue × Exp × Rating | 0.145 | 0.380 | 0.174 | 0.417 | 0.020 | 0.143 |
| Doctor profile: Intercept | 0.001 | 0.023 | 0.000 | 0.000 | 0.011 | 0.103 |
| Residual | 0.732 | 0.856 | 0.485 | 0.697 | 0.427 | 0.653 |

*Note.* N = 3676 (120 statements, 36 participants, 16 doctor profiles). "Abs" = absolute opinion change (|post − pre|); "Signed" = signed opinion change (post − pre); "Persu" = persuasiveness. Random effects: by-participant slopes for cue, rating, experience and their interactions; by-statement slope for cue; random intercept for doctor profiles. Correlations among random effects are available on request.
