# Supplementary material for: Neurocognitive mechanisms of source credibility: how work experience and patient ratings modulate the processing of medical veracity cues
Source: Front Hum Neurosci. 2026 Sep 18;20:1867901. doi: 10.3389/fnhum.2026.1867901 (PMC13630730; doi:10.3389/fnhum.2026.1867901)
Supplement: Supplementary file 6 [file Table_6.DOCX]

## **Table S6.** A linear regression model with ERP amplitude (150–210 ms), doctor’s credibility conditions (experience, rating), cue type and participants’ personality traits (CRT, NFCS, CS) predicting absolute opinion change

| **Predictor** | **β** | **SE** | **t** | **p-value** |
| --- | --- | --- | --- | --- |
| Intercept | 0.984 | 0.076 | 12.891 | *<0.001 |
| Amplitude | -0.017 | 0.012 | -1.373 | 0.171 |
| Cue (False vs True) | -0.372 | 0.083 | -4.493 | *<0.001 |
| Experience (Low vs High) | -0.197 | 0.082 | -2.386 | *0.018 |
| Rating (Low vs High) | -0.430 | 0.083 | -5.194 | *<0.001 |
| Cognitive Reflection | 0.094 | 0.055 | 1.694 | 0.091 |
| Need for Cognition | -0.039 | 0.059 | -0.656 | 0.513 |
| Conformity | 0.111 | 0.057 | 1.943 | 0.053 |
| Cue × Experience | 0.074 | 0.117 | 0.633 | 0.527 |
| Cue × Rating | 0.200 | 0.118 | 1.697 | 0.091 |
| Experience × Rating | 0.076 | 0.117 | 0.655 | 0.513 |
| Cue × Experience × Rating | 0.001 | 0.165 | 0.005 | 0.996 |

*Note.* Significant values are marked by *. Models were estimated by ordinary least squares on participant-by-condition means (36 participants × 8 condition means). Reference levels: cue type 'True', high work experience, high patient rating.
