# Supplementary material for: Neurocognitive mechanisms of source credibility: how work experience and patient ratings modulate the processing of medical veracity cues
Source: Front Hum Neurosci. 2026 Sep 18;20:1867901. doi: 10.3389/fnhum.2026.1867901 (PMC13630730; doi:10.3389/fnhum.2026.1867901)
Supplement: Supplementary file 7 [file Table_7.DOCX]

## **Table S7.** A linear regression model with ERP amplitude (150–210 ms), doctor’s credibility conditions (experience, patient rating), cue type ('True', 'False') and participants’ personality traits (CRT, NFCS, CS) predicting signed (directional) opinion change

| **Predictor** | **β** | **SE** | **t** | **p-value** |
| --- | --- | --- | --- | --- |
| Intercept | 0.562 | 0.195 | 2.884 | *0.004 |
| Amplitude | 0.000 | 0.013 | -0.020 | 0.984 |
| Cue (False vs True) | -1.365 | 0.099 | -13.824 | *<0.001 |
| Experience (Low vs High) | -0.123 | 0.098 | -1.260 | 0.209 |
| Rating (Low vs High) | -0.218 | 0.099 | -2.205 | *0.028 |
| Cognitive Reflection | -0.001 | 0.012 | -0.066 | 0.948 |
| Need for Cognition | 0.001 | 0.003 | 0.241 | 0.810 |
| Conformity | 0.002 | 0.002 | 0.948 | 0.344 |
| Cue × Experience | 0.283 | 0.138 | 2.049 | *0.041 |
| Cue × Rating | 0.539 | 0.140 | 3.865 | *<0.001 |
| Experience × Rating | 0.085 | 0.138 | 0.615 | 0.539 |
| Cue × Experience × Rating | -0.169 | 0.195 | -0.869 | 0.386 |

*Note.* Significant values are marked by *. Models were estimated by ordinary least squares on participant-by-condition means (36 participants × 8 condition means). Reference levels: cue type 'True', high work experience, high patient rating.
