# Supplementary material for: Neurocognitive mechanisms of source credibility: how work experience and patient ratings modulate the processing of medical veracity cues
Source: Front Hum Neurosci. 2026 Sep 18;20:1867901. doi: 10.3389/fnhum.2026.1867901 (PMC13630730; doi:10.3389/fnhum.2026.1867901)
Supplement: Supplementary file 8 [file Table_8.DOCX]

## **Table S8.** A linear regression model with ERP amplitude (150–210 ms), doctor’s credibility conditions (experience, rating), cue type and participants’ personality traits (CRT, NFCS, CS) predicting persuasiveness

| **Predictor** | **β** | **SE** | **t** | **p-value** |
| --- | --- | --- | --- | --- |
| Intercept | 5.674 | 0.191 | 29.778 | *<0.001 |
| Amplitude | -0.020 | 0.027 | -0.725 | 0.469 |
| Cue (False vs True) | -0.089 | 0.186 | -0.480 | 0.632 |
| Experience (Low vs High) | -0.667 | 0.185 | -3.599 | *<0.001 |
| Rating (Low vs High) | -1.486 | 0.186 | -7.982 | *<0.001 |
| Cognitive Reflection | 0.155 | 0.151 | 1.020 | 0.309 |
| Need for Cognition | -0.225 | 0.162 | -1.386 | 0.167 |
| Conformity | 0.090 | 0.156 | 0.577 | 0.565 |
| Cue × Experience | 0.044 | 0.262 | 0.167 | 0.868 |
| Cue × Rating | 0.092 | 0.265 | 0.349 | 0.728 |
| Experience × Rating | 0.236 | 0.262 | 0.900 | 0.369 |
| Cue × Experience × Rating | -0.016 | 0.371 | -0.043 | 0.966 |

*Note.* Significant values are marked by *. Models were estimated by ordinary least squares on participant-by-condition means (36 participants × 8 condition means). Reference levels: cue type 'True', high work experience, high patient rating.
